# A chromosome-scale genome assembly and epigenomic profiling reveal temperature-dependent histone methylation in iridoid biosynthesis regulation in *Scrophularia ningpoensis*

**DOI:** 10.1093/hr/uhae328

**Published:** 2025-03-04

**Authors:** Qing Xu, Chang Liu, Bin Li, Kewei Tian, Lei You, Li Xie, Huang Wang, Meide Zhang, Wuxian Zhou, Yonghong Zhang, Chao Zhou

**Affiliations:** Key Laboratory of Three Gorges Regional Plant Genetics and Germplasm Enhancement (CTGU)/Hubei Key Laboratory of Tumor Microenvironment and Immunotherapy, College of Biological and Pharmaceutical Sciences/College of Basic Medical Science, China Three Gorges University, Yichang 443002, China; Key Laboratory of Three Gorges Regional Plant Genetics and Germplasm Enhancement (CTGU)/Hubei Key Laboratory of Tumor Microenvironment and Immunotherapy, College of Biological and Pharmaceutical Sciences/College of Basic Medical Science, China Three Gorges University, Yichang 443002, China; Key Laboratory of Three Gorges Regional Plant Genetics and Germplasm Enhancement (CTGU)/Hubei Key Laboratory of Tumor Microenvironment and Immunotherapy, College of Biological and Pharmaceutical Sciences/College of Basic Medical Science, China Three Gorges University, Yichang 443002, China; Key Laboratory of Three Gorges Regional Plant Genetics and Germplasm Enhancement (CTGU)/Hubei Key Laboratory of Tumor Microenvironment and Immunotherapy, College of Biological and Pharmaceutical Sciences/College of Basic Medical Science, China Three Gorges University, Yichang 443002, China; Laboratory of Medicinal Plant, Institute of Basic Medical Sciences, School of Basic Medicine, Biomedical Research Institute, Hubei Key Laboratory of Wudang Local Chinese Medicine Research, Hubei University of Medicine, Shiyan 442000, China; Genome De novo Division, Wuhan Frasergen Bioinformatics Co., Ltd, Wuhan 430073, China; Genome De novo Division, Wuhan Frasergen Bioinformatics Co., Ltd, Wuhan 430073, China; Institute of Chinese Herbal Medicines, Hubei Academy of Agricultural Sciences, Enshi 445000, China; Institute of Chinese Herbal Medicines, Hubei Academy of Agricultural Sciences, Enshi 445000, China; Laboratory of Medicinal Plant, Institute of Basic Medical Sciences, School of Basic Medicine, Biomedical Research Institute, Hubei Key Laboratory of Wudang Local Chinese Medicine Research, Hubei University of Medicine, Shiyan 442000, China; Key Laboratory of Three Gorges Regional Plant Genetics and Germplasm Enhancement (CTGU)/Hubei Key Laboratory of Tumor Microenvironment and Immunotherapy, College of Biological and Pharmaceutical Sciences/College of Basic Medical Science, China Three Gorges University, Yichang 443002, China

## Abstract

Understanding how medicinal plants adapt to global warming, particularly through epigenetic mechanisms that modify phenotypes without changing DNA sequences is crucial. *Scrophularia ningpoensis* Hemsl., a traditional Chinese Medicine (TCM), produces bioactive compounds that are influenced by environmental temperatures, making it an ideal model for studying the biological basis of TCM geoherbalism. However, the adaptive potential of epigenetic marks in *S. ningpoensis* under varying temperatures remains understudied, partly due to the absence of a reference genome. Here, it was demonstrated that mild warm temperatures contribute to the metabolic accumulation and the cultivated migration of *S. ningpoensis* using a global dataset. A high-quality chromosome-level genome was assembled, and an atlas of epigenetic, metabolic, and transcriptomic profiles across different tissues. Transcriptome analysis identified 3401 allele-specific expressed genes (ASEGs) across nine tissues by comparing two haplotypes. ChIP-seq and BS-seq data from leaf and root tissues revealed that ASEGs are associated with distinct epigenetic patterns, particularly the active mark H3K36me3, which functions differently in these tissues. Notably, genes marked with H3K36me3 in iridoid synthesis pathway predominantly expressed in roots. Additionally, the histone methyltransferase *SnSDG8* was identified to regulate ectopic H3K36me3 in iridoid biosynthesis in response to warming temperatures. Our results highlight the epigenetic mechanisms of global warming on herb-derived products, significant for medicinal plant breeding under temperature stress.

## Introduction

Geoherbalism, also known as ‘Daodi’ in China, refers to Traditional Chinese Medicine (TCM) grown in specific native environments, where they are believed to produce higher quality medicinal properties due to environmental influences on their metabolic products, compared to those cultivated elsewhere [1,2]. Among various environmental factors, global climate change is a major force affecting agricultural ecosystems, including the metabolic processes in medical plants [3,4]. As greenhouse gas emissions rice, global warming has altered the geographical distribution of herbs [5,6], leading to the migration of TCM cultivated regions. For instance, the cultivated regions of wild jujube are expected to expand and become naturalized in northwest Chinas [7], while suitable habitats for Southern prickly ash are predicted to decline [8]. Although the high quality of Daodi herbs is closely linked to their growing environments [9–11], how they respond to global warming remains largely unexplored.

In model plants and crops, environmental stresses often trigger changes of epigenetic signatures, such as DNA methylations and histone marks [12,13]. For example, in *Arabidopsis*, mild heat stress impacted seed development and triggered dynamic changes in DNA methylations during germination [14]. In cultivated *Brassica napus* microspores, brief heat shock treatment of caused widespread DNA hypomethylation [15], while in soybean, heat exposure similarly led to DNA hypomethylation, particularly at CHH sites [16]. In contrast, a heat-resistant cotton cultivar showed DNA methylation increases, whereas a sensitive cultivar experienced significant DNA CHH methylation disruption [17]. Histone marks also changed in response to heat in plants. For instance, warming stress reduced the levels of H3K9me2 in *Arabidopsis* and cork oak [18,19], while high temperatures increased the levels of H3K4me2 and H3K9ac in maize promoters [20]. These findings suggest that the effects of warming on histone marks vary across plant species. In *Arabidopsis*, the inactivity of H3K4 methyltransferases, such as SET domain group25 (SDG25) and *Arabidopsis* homolog of trithorax1, resulted loss of H3K4me3, thereby repressing heat-induced genes [21], while mutants of the *Arabidopsis histone deacetylase 19* showed enhanced heat resistance [22]. H3K36me3, a key epigenetic mark, influenced flowering time in response to ambient temperature [23], a process controlled by the histone methyltransferase gene *SDG8*, with its mutant *sdg8* showing reduced H3K36me3 enrichment [24]. In the alpine herb *Wahlenbergia ceracea*, temperature-induced epigenetic variation increased in low-elevation seedlings [25]. However, comprehensive studies on epigenetic regulation in medicinal herbs remain limited.

*Scrophularia ningpoensis* Hemsl., a temperature-sensitive plant, thrives in regions with relatively higher average temperatures and lower altitudes [26], making it an ideal model for studying plant adaptation as well as TCM geoherbalism. Its dried roots, known as Radix Scrophulariae, have been used as TCM over 1000 years [27]. The plant’s iridoids, known for anti-inflammatory and many other therapeutic properties, are highly bioactive [28,29]. Some species within the family Scrophulariaceae have been documented for ploidy and genome size, demonstrating that most closely related species, including *S. ningpoensis*, are diploid with genome sizes typically under 1 Gb [30]. Given the absence of genome assembly and limited epigenomic research on *S. ningpoensis* [31,32], this information provides a valuable reference for initialing genomic studies in *S. ningpoensis* in this study.

Herein, to probe the role of epigenome modulation in plant responses to warming stress, a chromosome-scale genome assembly with haplotype resolution was performed for *S. ningpoensis.* The epigenome and metabolome in the leaf and root tissues were analyzed, coupled with transcriptomic data across different developmental stages. Using PacBio long-read sequencing combined with genomic Hi-C interaction data and an optimized genome assembly process, a comprehensive genome of approximately 730 Mb was successfully reconstructed. All sequences were accurately mapped onto 92 pseudochromosomes, yielding a contig N50 of 14.58 Mb. This high-quality genome allowed for integrative profiling of DNA methylation, nine histone modifications, and metabolome from the leaf and root tissues, along with transcriptomes from nine different tissues. Comparisons of the two haplotypes revealed 70 614 allelic genes, with those exhibiting high expression levels preferentially enriched by H3K27ac, H3K4me3, and H3K36me3. Analyzing chromatin states (CSs) based on their combinatorial patterns indicated that the transcriptional regulation of H3K36me3 may have distinct functional rules in leaf and root tissues, particularly concerning iridoids biosynthesis. Additionally, a histone methyltransferase gene *SnSDG8* may contribute to iridoids synthesis by writing H3K36me3 in *S. ningpoensis* under warming. This study provides novel insights into the epigenetic modulation of warming stress responses in *S. ningpoensis*, a species known for its resilience to environmental changes.

## Results

### Climate warming favors the cultivation of Daodi *S. ningpoensis*

**Figure 1:**
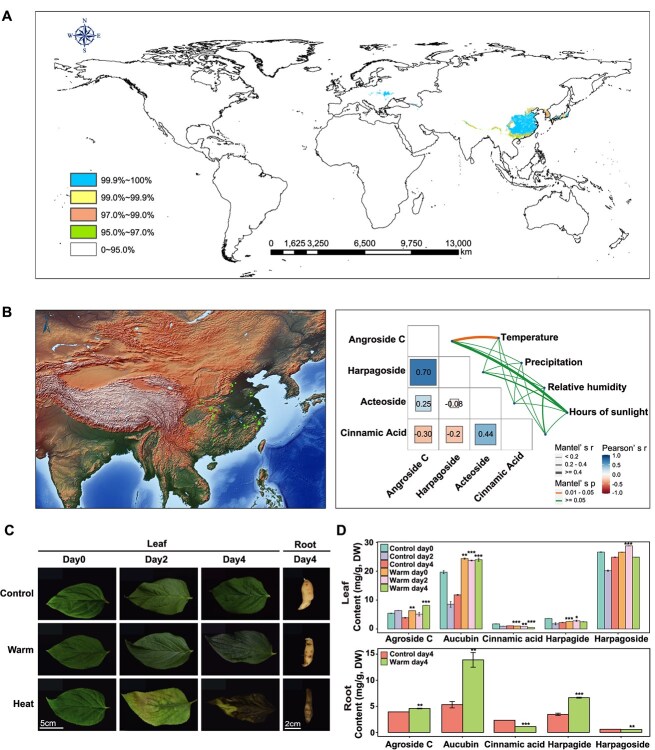
Iridoids dynamic is positively associated with temperature in *S. ningpoensis*. (**A**) Potential cultivated region predicted for *S. ningpoensis* according the GMPGIS. (**B**) The sampling points of *S. ningpoensis* in China are shown in the left panel. The red dots represent the sampling points. Pairwise comparisons of the ambient-level distribution of typical metabolites in *S. ningpoensis* are shown in the right panel, with a color level that refers to Pearson's correlation coefficients. Environmental factors (temperature, precipitation, relative humidity, and hours of sunning) are correlated with the content of metabolites (angroside, harpagoside, acteoside, cinnamic acid) with the Mantel test. The width of the edge reflects Mantel's r statistic for the distance correlations, while the color indicates the level of statistical significance. (**C**) Growth status of *S. ningpoensis* under different temperatures (control: 28°C; warm: 35°C; heat: 42°C). (**D**) The contents of five components in dry leaves and roots under different temperatures. The average ± SD values from three biological repeats are shown. Statistical significance was determined using *t*-test; ***P < 0.001, **P < 0.01, *P < 0.05.

Given the close association between plant adaptation, biodiversity, and global climate change [33], evaluating how *S. ningpoensis* responds to climate shifts across its global population is crucial. With the help of Geographic Information System for Global Medicinal Plants (GMPGIS), a model was developed to identify ecologically cultivated regions of medicinal plants [34]. A total of 265 sampling points worldwide were analyzed (Fig. S1; Table S1). The analysis revealed that ecologically cultivated regions for *S. ningpoensis* are primarily located in East Asia (China and other countries), with some areas in Central and Eastern Europe (Fig. 1A), consistent with known distribution [35]. Under projected global climate change scenarios, potential suitable cultivation areas for *S. ningpoensis* are expected to see minimal increases by 2070 compared to current ecologically suitable areas, with potential for shift or migrate in cultivation regions (Fig. S2). As higher average temperatures and lower altitudes are recommended for *S. ningpoensis* cultivation under Good Agricultural Practices [26], a Mantel test was conducted to assess the correlation coefficient between environmental factors and iridoid production. Angroside C showed a particularly strong association with temperature (Fig. 1B). To validate these findings, controlled experiments simulating temperature increases were performed. Results indicated that moderate temperature increases enhanced iridoids biosynthesis, while excessively high temperatures caused rapid plant apoptosis (Fig. 1C and D). Overall, climate warming may drive the migration of Daodi *S. ningpoensis* cultivation regions. To ensure high-quality crude medicine production, it is essential to assess the impact of temperature on plant growth to optimize growing conditions for *S. ningpoensis*.

### *De novo* genome assembly and annotation of *S. ningpoensis*

**Figure 2:**
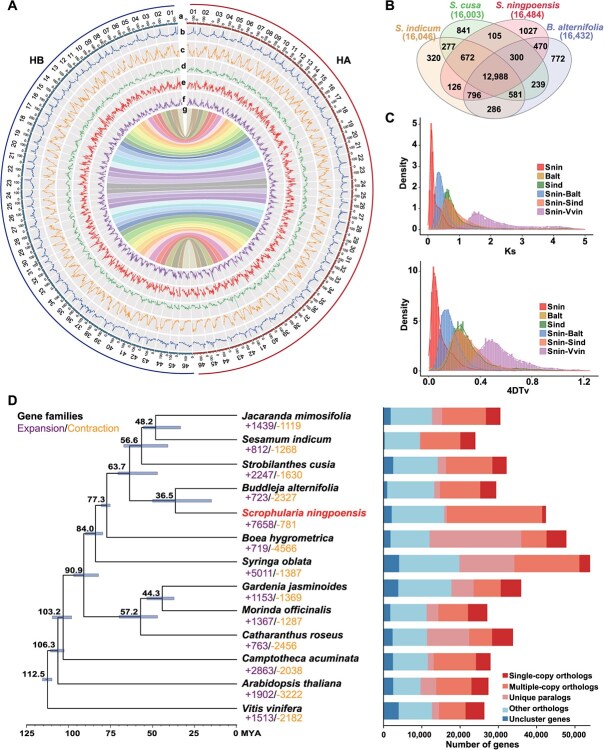
*De novo* genome assembly and phylogenetic analysis of *S. ningpoensis*. (**A**) The haplotype-resolved genome assembly of *S. ningpoensis*. Genomic traits of the haplotypic resolved *S. ningpoensi*s genome for HA (in red) and HB (in blue) are shown in the tracks. The various genomic features are represented as follows: a stand for chromosome length (Mb). b represents the GC content. c-d represents the density of genes, repeat, Copia elements, Gypsy elements, links between the haplotypes. All calculations were done within 500 Kb windows. (**B**) Shared and unique gene families among *S. ningpoensis* and other three plants. (**C**) Synonymous nucleotide substitution (Ks) and 4-fold synonymous (degenerative) third-codon transversion (4DTv) revealed WGD event in the evolutionary processes of *S. ningpoensis*. (**D**) Phylogeny tree for *S. ningpoensis* and twelve others. The estimated variance time is shown at each node; bars are 95% fiducially interval. Gene family expansions are indicated in purple, and gene family contractions in yellow. Genes of *S. ningpoensis* and other species are categorized into five classes (single-copy orthologs, multiple-copy orthologs, unique paralogs, other orthologs, and uncluster genes).

Before examining how temperature affects the active ingredients in *S. ningpoensis*, it was sequenced a cultivated *S. ningpoensis* individual, expecting to provide a multiomics perspective. Cytogenetic analysis confirmed a diploid genome (2*n* = 2*x* = 92) for the testing plant (Fig. S3). A total of 341.59 Gb of high-quality Illumina reads and 68.45 Gb of PacBio HiFi reads with an N50 of 15 kb were generated, along with 160.45 Gb of Illumina Hi-C sequencing data (Tables 1 and S2). The genome size was estimated at 720–730 Mb by flow cytometry (Fig. S4A and B) and 787 Mb by K-mer analysis (*K* = 19), with a heterozygosity rate of 2.82% (Fig. S4C). *de novo* assembly using Hifiasm produced a 1.57 Gb genome assembly, consisting of 864 contigs with an N50 length of 14.58 Mb. Following the integration of Hi-C data anchoring, 1.47 Gb of the sequence was organized into 92 pseudochromosomes (Fig. S5), ranging from 10.36 to 24.43 Mb in length (Table S3). Although our computational pipeline does not use a specific assembly procedure or configuration for a stepwise assembly approach, it was obtained a collection of 46 homologous chromosome pairs in diploid *S. ningpoensis* at the chromosome scale (Fig. 2A). Without parental information, homologous chromosomes were assigned as haplotype A (HA) and haplotype B (HB) based on the Hi-C mapping data (Fig. 2A). Totally, approximately 94.08% of fragments were mapped to pseudochromosomes in the two haplotypic chromosomes. The ultimate assembled genome size was 731.44 Mb for HA and 735.13 Mb for HB (Fig. 2A; Table 1). Using various validated methods, it was confirmed a superb reference assembly for the *S. ningpoensis* genome (Table S4). It was shown that 1599 (99.0%) and 1592 (98.6%) of 1614 universal single copy embryophyte genes could be found from HA and HB, respectively, with Benchmarking Universal Single-Copy Orthologs (BUSCO) (Table S5).

**Table 1:** Summary of genome assembly and annotation of *S. ningpoensis*.

Sequencing	*Scrophularia ningpoensis*	
Illumina sequencing		
Clean data (Gb)	341.59	
Sequencing depth (X)	434.40	
PacBio sequencing		
HiFi reads (Gb)	68.45	
Sequencing depth (×)	86.97	
Average reads length (bp)	14 679	
Reads N50 (bp)	15 088	
Hi-C sequencing		
Clean data (Gb)	160.45	
Sequencing depth (×)	203.29	
Genome survey		
Estimated genome size (Mb) per 1 C	787.05	
Heterozygous ratio (%)	2.82	
Repeat (%)	68.70	
Haplotype-resolved chromosomal-level assembly and annotation		
	HA	HB
Assembly size (Mb)	731.77	735.13
BUSCO completeness of assembly (%)	99.0	98.6
BUSCO completeness of annotation (%)	96.5	95.6
Number of annotated genes	42 421	41 972
Number of genes with annotated alleles	35 307	35 307

A comprehensive approach, combining *de novo*, homology-based, and transcriptome-based predictions, was used to annotate protein-coding genes (PCGs) in the S. ningpoensis genome. This strategy identified 89 073 PCGs, with 84 393 (94.75%) successfully mapped to 92 chromosomes (Table 1). BUSCO indicated that 96.5% and 95.6% of the annotated genes possessed the whole sequence information from HA and HB (Table S5). It also predicted noncoding RNA genes, identifying 613 microRNA genes, 2934 transfer RNA genes, 13 833 ribosomal RNA genes, and 3649 small nuclear RNA genes (Table S6). Additionally, repetitive sequences were identified in both haplotypes, comprising 508.00 Mb in HA (69.45%) and 501.29 Mb in HB (68.19%). Long terminal repeat (LTR) retrotransposons accounted for 42.11% of HA and 41.12% of HB, with the Copia and Gypsy superfamilies comprising 27.31% and 27.29%, and 14.37% and 13.36%, respectively (Table S7).

Next, a comparative analysis of PCGs in *S. ningpoensis* with three other Lamiales species (*Buddleja alternifolia*, *Strobilanthes cusia*, and *Sesamum indicum*) revealed 12 988 common gene families and 1027 families specific to *S. ningpoensis* (Fig. 2B). The Ks values of the collinear gene pairs ranged from 0.10 to 0.13, indicating whole-genome duplication (WGD) events occurred approximately 8.92–11.61 million years ago (Mya). Two additional WGD events were identified in *S. ningpoensis*: one occurring 28.57 to 33.04 Mya (Ks: 0.32–0.37), in the *Scrophulariaceae* lineage, and the another 72.32 to 75.35 Mya (Ks: 0.81–0.84) shared with *B. alternifo*lia and *S.* indicu*m* (Fig. 2C).

A phylogenetic tree was constructed using 191 single-copy gene families across 13 species, revealed that *S. ningpoensis* and *B. alternifolia*, positioned at the base of the asterids, diverged from the euasterids around 63.7 Mya ago and from each other around 36.5 Mya (Fig. 2D), indicating a closer genetic relationship between these two species compared to others. Expansion and contraction analysis identified 7658 expanded and 781 contracted families in *S. ningpoensis*. The enrichment analysis showed that the expanded families were associated with response to environmental factors, such as GO:0009415 (response to water), while the contracted families were associated with metabolite biosynthesis, such as GO:0044550 (secondary metabolite biosynthetic process) (Table S8).

### Haplotypic diversifications and allelic imbalance

**Figure 3:**
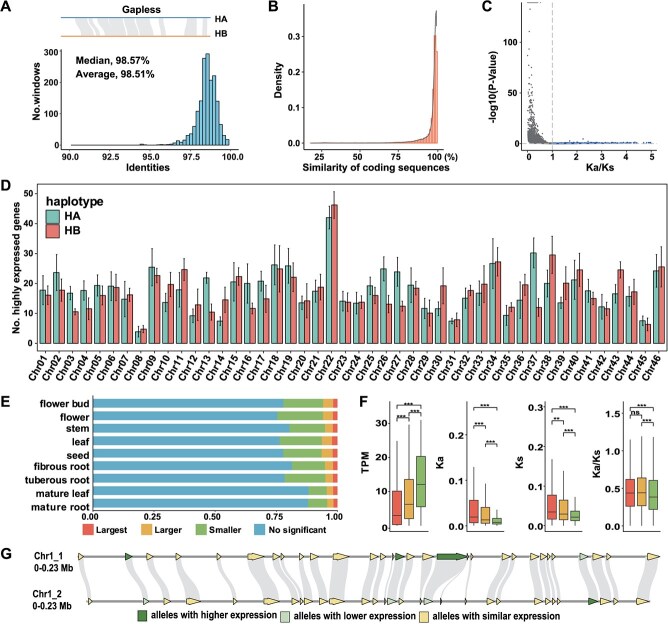
Allelic imbalance between *S. ningpoensis* haplotypes. (**A**) A comparison of two haplotypic entire genomes was conducted within windows of 200 Kb that did not overlap, allowing for no extension gaps within alignment blocks. The median and average were calculated based on the identities of all Windows. The lower panel displayed the distribution of similarities, where the *x*-axis was similarities and the *y*-axis were the number of windows supporting each similarity. (**B**) The coding sequences of alleles were compared pairwise. (**C**) The distribution of Ka/Ks values for allelic genes was compared pairwise. Genes with Ka/Ks > 1 were represented by blue dots, while genes with Ka/Ks < 1 are represented by grey dots. The statistical significance was determined using a two-sided Fisher's exact test to calculate *P* values. (**D**) Quantitative analysis of genes exhibiting high expression levels on haplotype chromosomes across nine distinct tissue types. The graphs illustrate the mean numbers of such genes along with the standard deviation ± SD. (**E**) Allelic expression bias proportions categorized per allele across the nine tissues. The following categories of allelic expression bias were defined: (1) no significant allele expression differences (*P* ≥ 0.05), (2) smaller allele expression differences with a fold-change (FC) ≤ |2| (*P* < 0.05), (3) larger allele expression differences with a |2| < FC < |8| (*P* < 0.05), and (4) largest allele expression differences with FC ≥ |8| (*P* < 0.05). (**F**) Absolute abundance of transcripts per million, along with the Ka, Ks, and the Ka/Ks ratios for alleles with varying degrees of expression differences: minimal, moderate, and maximal (*P < 0.05). Colors (largest, larger, and smaller) are consistent with cluster colors in (**E**). (**G**) Detailed syntenic block view highlighting the alleles with higher expression (dark green) versus those with reduced expression (light green) on both haplotypes.

High levels of heterozygosity enable the acquisition of phase II haplotypes through ALLHiC [36]. Haplotype phasing produced 1.47 Gb of haplotype-resolved sequences, mapped to 92 pseudochromosomes (Table S9). To probe sequence differences and evolutionary associations, the genome sequences were rigorously mapped, allowing gaps or subscripts in the alignment blocks. This analysis revealed that average sequence identity between the two haplotypes was 98.52% (Fig. 3A). A total of 2.76 million SNPs, 220 704 insertions, and 230 941 deletions were identified (Table S10), spanning 1.39% (10.16 Mb) of the compiled monoploid genetic material. Both haplotypes displayed comparable amounts of transposon components, with 69.45% in HA and 68.19% in HB (Table S10). Polymorphisms were also identified among the 46 homologous chromosome pairs, allowing accurate divergence assessment the between the haplotypes (Figs S6 and S7). Utilizing MCscanX [37], a total of 70 614 allelic genes at 35 307 loci (83.67%) were identified between the HA and HB pairs (Table S11), with highly similarity (mean identity: 96.35%; Fig. 3B). Most allelic genes undergo purifying selection, reflected by the of Ka/Ks ratio of 0.39 (Fig. 3C). About, 46 902 structural variations (SVs) were recognized between HA and HB using SyRI [38] (Table S12). These differences highlight structural and functional allelic differences in our haplotype-phased *S. ningpoensis* assembly.

Subsequently, allele-specific expression (ASE) was investigated without parental genome information (Table S13). The identified ASE genes (ASEGs) exhibited no significant differences between allelic chromosome pairs, with a few isolated exceptions (Fig. 3D). This implies that, in general, the gene expression was unbiased between two haplotypes. By analyzing gene expression patterns observed in nine distinct tissues (refer to Table S14), rigorous categories for allelic expression bias were established. Most alleles (75.42%–88.23% of alleles, Fig. 3E) exhibited no substantial transcriptional variation events within the same tissues, with consistent expression differences seen in only 1.62%–2.22% of allelic sites across various tissues or organs (Fig. 3E). Moreover, allelic sites with minimal ASE differences had higher absolute transcriptional compared with those in other classifications (Fig. 3F). In contrast, the category with the greatest expression differences contained alleles with notably higher rates of nonsynonymous mutations (Ka), synonymous mutations (Ks), and Ka/Ks ratios when compared to other categories (Fig. 3F). These results revealed that the alleles exhibited sequence variation and it may underlie differences in allelic expression. Notably, ASEGs were scattered randomly across the genome, with alternating active and inactive allelic sites present in HA and HB (Fig. 3G). Additionally, ASEGs exhibited enrichment in biological functions, for instance, the behavior to stimuli (Fig. S8), unlike genes with large-effect variations (Fig. S9). Allele-specific expressed genes are known to contribute to temperature stress resistance [39,40]. Genetic crosstalk between alleles plays a significant role in plants with simple diploid genomes [41], though the regulatory mechanism governing allelic gene expression in highly heterozygous diploids remains unclear. These finding led us to hypothesize that allelic imbalance might serve as a potential mechanism for adapting to climate temperature changes.

### Epigenomic profiling of leaf and root tissues in *S. ningpoensis* genome

To investigate the epigenomic characteristics and elucidate the regulatory mechanisms of ASE genes in *S. ningpoensis*, nine histone signatures were generated using the ChIP-seq approach, coupled with DNA methylome and transcriptomes from leaf and root tissues (Table S15). The two replicates of ChIP-seq data exhibited strong Pearson correlation coefficients (Fig. S10A). Similar trends were observed in the methylomes (Fig. S10B) and transcriptomes (Fig. S10C). These results demonstrated high reliability and reproducibility of our data. Additionally, the consistency of these ChIP-seq, BS-seq, and RNA-seq datasets was further demonstrated by their representative patterns in a ∼ 118 kb chromatin region (Fig. S10D).

Acetylation of histones at various lysine locations, referred to as active histone modifications, is concentrated surrounding the transcription start site (TSS) of expressed genes [42]. This pattern has also been observed in other active modifications, such as H3K4me3 and H3K36me3. The active marks H3K9ac and H3K27ac are prominently presenting in intergenic regions and mark active enhancers (Fig. S11A and Fig. S12; Table S16). In contrast, inactive histone marks, such as H3K9me2 and H3K27me2 were found in higher proportions within transposable element genes or repeats (Fig. S11B and Fig. S12; Table S16). These marks were predominately found in regions within the transcriptional body (Fig. S11A). The overall levels of DNA methylation in *S. ningpoensis* were around 25.15% and 27.93% in leaf and root tissues, respectively. Within three contexts of CG methylation, CHG methylation, and CHH, methylated ratios were approximately 81.07%, 54.23%, 10.37% in leaf and 81.25%, 55.16%, 14.01% in root, respectively (Fig. S11C). These percentages can vary among different plant species [43–46]. At the chromosomal scale, methylation of CG and CHG types is predominantly found in the heterochromatic regions; whereas methylation of the CHH type is typically localized in euchromatic regions (Fig. S11D). Genes associated with transposable elements (TEs) exhibit heavy CGme and CHGme, leading to the suppression of their transcriptional activity (Fig. S11E and S12). In the case of PCGs, CHGme and CHHme levels were minimal within transcriptional regions; however, CG methylation within transcriptional regions was positively associated with high expression (Figs S11E and S12). Collectively, these findings regarding the histone mark profiles and DNA methylations in the *S. ningpoensis* genome align with results from previous studies in other plant species [44,47–51].

### Asymmetrical epigenomes and transcriptomes cause haplotype imbalance in mature roots of *S. ningpoensis*

**Figure 4:**
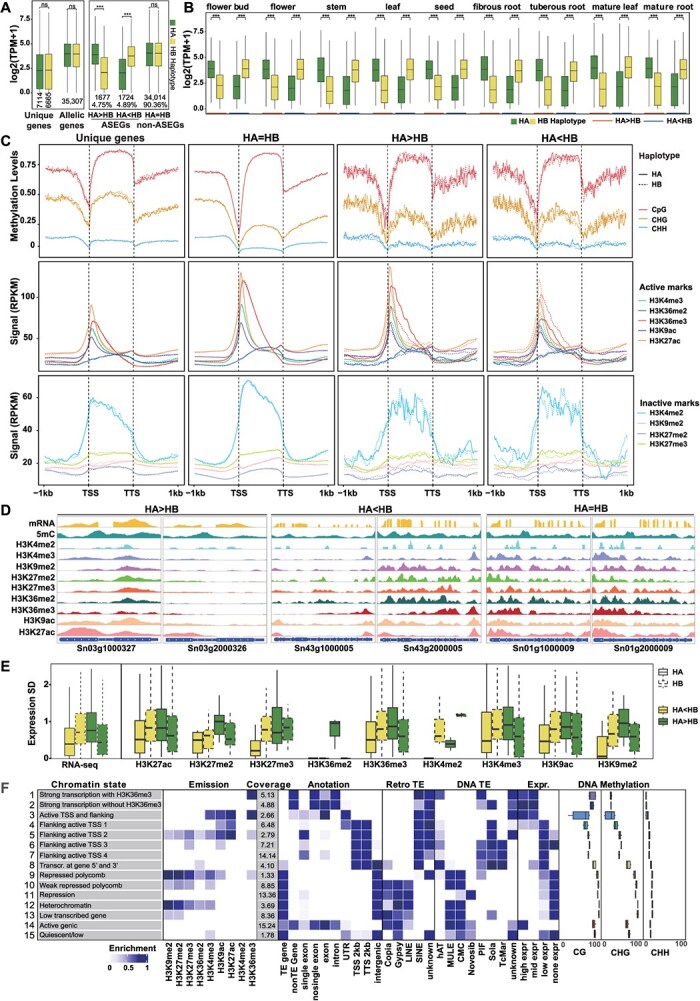
Asymmetrical distribution of epigenomic marks in HA chromosomes and in HB chromosomes in root tissue of *S. ningpoensis* genome. (**A**) Expression levels of haplotype-unique and allelic genes in the HA and HB chromosomes (left). According to allelic genes’ transcriptional levels in nine tissues, ASEGs were divided into two categories, HA > HB and HA < HB. When HA = HB, these allelic genes have no differences in transcriptional levels (non-ASEGs). The quantities of genes within a specific classification are displayed. Utilizing the Wilcoxon rank-sum test, the statistical significance of the variances was assessed, ***P < 0.001. (**B**) Levels of expression for genes expressing allelic specificity on the HA and HB chromosomes. (**C**) The levels of DNA methylation, occupancy of histone modifications in the genic regions of the HA and HB genomes. (**D**) Demonstrative instances of pairs of genes expressing allelic specificity in (C) are presented. (**E**) The variation in transcription, DNA methylation, and occupancy of histone modifications of genes expressing allelic specificity between the HA and HB genomes. (**F**) The meanings of CSs, abbreviations, and composition (emission probability) of histone marks and occupancy of DNA methylation.

Despite the absence of significant differences in transcription expression between Haplotype-unique genes (namely ‘unique genes’) or allelic genes in the *S. ningpoensis* genome for HA and HB, it was observed a notable expression bias (fold change >2, *P* < 0.05) was observed in homologous gene pairs derived from defined ASEGs (Fig. 4A) across all samples. Specifically, 4.75% (1677) of these gene pairs exhibited higher expression in HA compared to HB (HA > HB), while 4.89% (1724) showed lower expression in HA than in HB (HA < HB). The majority, comprising 90.36%, displayed equal expression in both haplotypes (HA = HB) (Fig. 4A). Detailed analysis revealed that this asymmetrical transcription between the two haploids was primarily owing to the various expressions of allelic genes and was unrelated to tissue type (Fig. 4B). Additionally, the data revealed a preferential enrichment of H3K27ac, H3K4me3, and H3K36me3 on the side with dominant expression in ASEGs (Fig. 4C). Accordingly, when the transcriptional level of ASEG much more active in HA, these marks are also more enriched, and vice versa (Fig. 4C and D). However, this pattern was not observed in haplotype-unique genes and allelic gene pairs with equal expression level (Fig. 4C and D). Furthermore, an imbalance in transcription and dynamics of chromatin signatures was identified in ASEGs from the HA and HB genomes in root tissue (Fig. 4C). These dynamic transcriptional levels can be attributed to varying levels of histone marks, excluding DNA methylation, between the two haplotypes (Figs 4E and S13). Correspondingly, it was noted that the variability in the occupancy of histone marks (H3K27ac, H3K36me2, H3K36me3) was obviously higher in genes of HA > HB were compared to those of HB < HA (Figs 4E and S14B). Notably, our observation in *S. ningpoensis* was inconsistent with a previous result in potato plants, which suggested that nearly one-third of tomato allelic gene pairs exhibited differential expression and methylated loci between alleles [52]. These findings suggest that the imbalanced gene expression between the HA and HB haplotype-resolved genomes in *S. ningpoensis* is, at least partially, linked to the distinct epigenetic status of the two haplotypes.

To further elucidate the epigenetic marks that play a key role in ASEGs, CSs were characterized according to their integrated modes across multiple epigenomes. CS maps have been proven to represent gene annotation and transcriptional activity [53–55]. Thus, using ChromHMM v.1.24 software, it was aimed for 15-CS models at a 200 bp resolution in the two tissues from *S. ningpoensis* (Figs 4F and S14C). Consistent with previous analysis, the heterochromatin state (Het/CS12), ruled by H3K9me2, substituted for 3.69% in genome and was involved in heavy DNA methylation, enriched transposons, and silenced transcription. Another distinct state, the quiescent state (CS15), lacking of any surveyed epigenetic signatures, substituted for intergenic regions and highly enriched in the Gypsy retrotransposon and DNA transposons (Fig. 4F). The repressive states with low or none expressed levels, including repressed Polycomb/CS9 and weak repressed Polycomb/CS10, were occupied by H3K9me2, H3K27me2, and H3K27me3 (Fig. 4F). These repressed states, predominantly associated with Polycomb, were largely observed in intergenic regions, substituted for 10.18% of the genome, and was linked to highly enriched transposon elements and heavy cytosine methylation (Fig. 4F). In contrast, transcriptional states displayed lowly enriched for retrotransposons and DNA transposons (Fig. 4F). Similarly, inactive states (flanking active at TSS/CS4-CS7, transcribed at gene 5′ and 3′/CS8, low transcribe gene/CS13) appeared to be associated with low and no transcriptional activity and were rich in retrotransposons (Fig. 4F). Notably, top active states (high- and mid-expressed level) covered, on average, 27.91% of the reference epigenome and consisted of active transcriptional events (strong transcription with H3K36me3/CS1, strong transcription without H3K36me3/CS2, active TSS and flanking/CS3, active genic/CS14) (Fig. 4F). These active states commonly had a higher frequency of H3K36me3, low enrichment for TEs, and relatively high CG methylation, covering 5.13% of the genome (Fig. 4F). In crop epigenomes, including rice and sorghum, moderate gene body CG methylation with active transcriptional events has been observed [48,49]. Interestingly, this combination of CSs was not found in leaf tissue (Fig. S14C). Overall, these CSs could be broadly classified into four categories: active state, repressive state, inactive state, and quiet state. With the exception of the silent status, about 98.22% from the genome of *S. ningpoensis* was identified as minimal epi-marks. Various status display unique coverage, transcription, transoms, and cytosine methylation (Fig. 4F). Notably, these findings highlight the links between epigenetic signatures and asymmetrical gene expression of HA and HB, in particular the differential roles of H3K36me3 in the transcriptional events of both homologous gene pairs and haplotype-unique genes.

### Global H3K36me3 dynamics and metabolic landscapes in leaves and roots of *S. ningpoensis*

**Figure 5:**
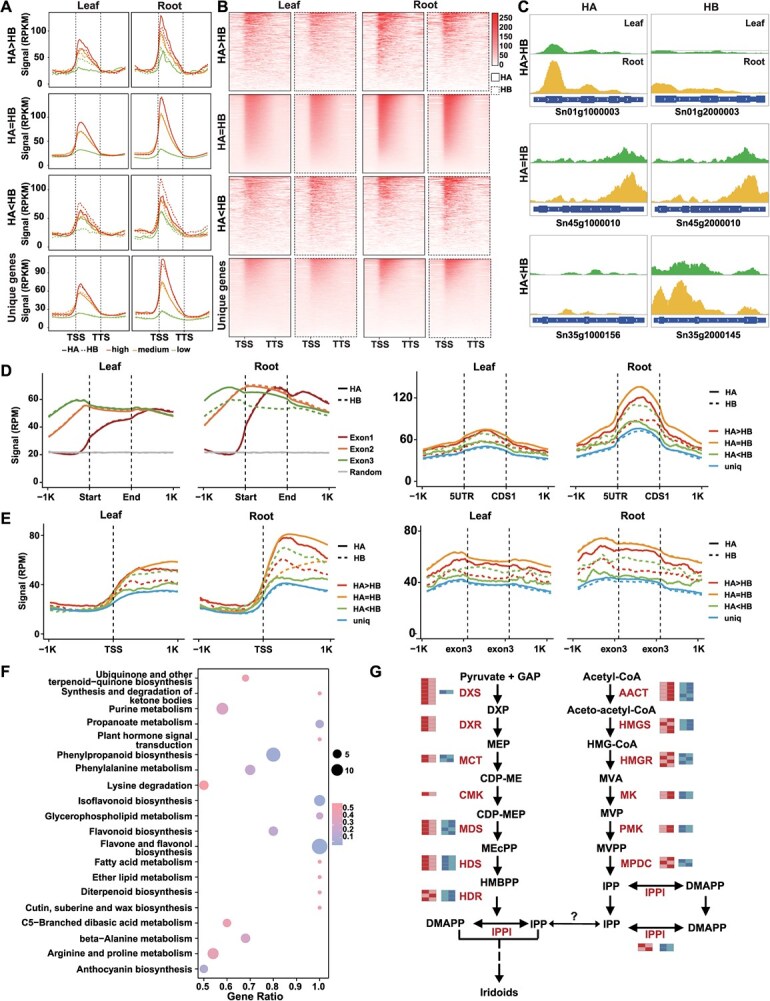
Dynamic of histone mark, gene expression and metabolic product in leaf and root tissues from *S. ningpoensis*. (**A**) Curve plots depicting the distribution of H3K36me3 signals extending from 1 kilobase upstream of the TSS to 1 kilobase downstream of the transcription end sites. Genes, both ASEG and non-ASEG types, were categorized into five distinct bins according to their FPKM values: 0, 0–1, 1–10, 10–20, and greater than 20. (**B**) Heatmap illustrating the distribution of H3K36me3 read counts ranging from 1 kilobase upstream of the TSS to 1 kilobase downstream of the TESs. These genes were sorted in descending order based on their FPKM values. The color gradient transition from black to yellow indicates H3K36me3 read intensities, ranging from low to high levels. (**C**) Representative H3K36me3 enrichments related with ASEGs and non-ASEGs, respectively. (**D**) Distribution of H3K36me3 enriches level in the first three exons (Exons 1, 2, and 3), 5′ UTR/CDS, Exon3 and ± 1 kb flanking in the two tissues. (**E**) Assignment of H3K36me3 signal level within ±1 kb of TSSs in leaf and root. **(F)** KEGG pathway enrichment analysis of DAMs. (**G**) Simplified representation of transcriptional and H3K36me3 of genes involved in iridoids biosynthetic pathway in leaf and root. Red represents gene expression and blue represents gene H3K36me3 signal level. Light and deep color represents low and high level, respectively. DXS: 1-deoxy-D-xylulose-5-phosphate synthase; DXR: 1-deoxy-D-xylulose 5-phosphate reductoisomerase; MCT: MEP cytidyltransferase; CMK: CDP-ME kinase; MDS: 2-C-methyl-D-erythritol 2,4-cyclodiphosphate synthase; HDS: 1-hydroxy-2-methyl-2-butenyl 4-diphosphate synthase; HDR: HMBPP reductase; AACT, acetoacetyl-CoA thiolase; HMGS, 3-hydroxy-3-methylglutaryl-CoA (HMG-CoA) synthase; HMGR: 3-hydroxy-3-methylglutaryl-CoA synthase; MK, mevalonate kinase; PMK, phosphomevalonate kinase; MPDC, diphosphomevalonate decarboxylase; IPPI, isopentenyl diphosphate Delta-isomerase.

To examine the dynamics of H3K36me3 and its potential roles in gene expression states in *S. ningpoensis*, it was methodically analyzed the connection between various epigenetic signatures and transcription in leaf and root tissues (Table S17 and Table S18). A strong positive association was observed between H3K36me3 intensities and gene expression changes in both tissues, along with a compact association between H3K36me3 levels and gene expression alterations in both tissues. Additionally, it was observed a robust positive association between H3K36me3 and active marks like H3K4me3, H3K9ac, and H3K27ac (Fig. S15). Undoubtedly, genes marked with H3K36me3 exhibited high transcriptional activity compared to random genes (Fig. S16), suggestion a regulatory role of H3K36me3 (refers as CS1) in the *S. ningpoensis* genome. H3K36me3 was predominantly found in promoter and 5′ untranslated sequences (UTRs), but with lower levels found in intron and 3′UTR, correlating positively with transcriptional activity in both leaves and roots (Fig. S17). Subsequently, genes with H3K36me3 were categorized into three bins based on their transcriptional activities from the two tissues and H3K36me3 was distributed in the flanking regions of these genes (Fig. 5A). From an evolutionary perspective, the conserved active mark H3K36me3 in *S. ningpoensis* was also abundant near TSS (Fig. 5A). A heatmap displaying H3K36me3 levels in different genic regions, ranked by expression levels (FPKM), further illustrated this genic distribution pattern (Fig. 5B). Interestingly, root tissue showed a preference for recruiting H3K36me3 marks compared to leaf tissue, suggesting a tissue-specific role for H3K36me3 (Fig. 5A and B). Additionally, the pattern data presented here supported that H3K36me3 occupancy was higher in ASEGs where HA > HB, and lower where HA < HB (Figs 4E, 5A and B). Representative H3K36me3 distributions for homologous gene pairs (HA > HB, HA = HB and HA < HB) and haplotype-unique genes in leaf and root tissues are illustrated (Fig. 5C). Overall, our analysis strengthened that H3K36me3-mediated gene transcriptional regulation is associated with its own CSs in the two tissues in the *S. ningpoensis*.

To further explore the distribution of H3K36me3 across various transcriptional regions, it was distributed normalized H3K36me3 signals of the first three exons. In relation to exon 1, exon2, and random control, H3K36me3 reads were significantly enriched in the third exon in root compared to leaf tissue (Fig. 5D). Normalized H3K36me3 read counts in both leaf and root tissues were subsequently plotted for exon 1 (5′ UTR and CDS1) (Fig. 5D), exon 3, and TSS (Fig. 5E), revealing higher contribution from H3K36me3-marked allelic gene (HA > HB, HB < HA) in the root (Fig. 5D and E). A total of 17 386 genes were identified transitioning from H3K36me3-marked ASEGs in the root to active states in the leaf. KEGG enrichment analysis indicated that these genes are primarily taken part in the secondary metabolic products synthesis, including terpenoid, phenylpropanoid, and purine (Fig. 5F). These results suggested that H3K36me3 rules in a gene position-dependent manner to modulate metabolic synthesis by enhancing transcriptional activities in *S. ningpoensis*, with more noted pronounced effects in the root tissue, the medicinal parts, implying distinct tissue-specific functional roles.

To assess global metabolic flux in leaf and root tissues, it was collected both the same tissues and conducted metabolic profiling using HPLC-Q-TOF/MS (Fig. S18A). Altogether, analysis detected 683 metabolites that aligned with established biochemical coefficients (Table S19). The high reproducibility of these findings is evidenced by the strong Pearson correlation coefficients observed among triplicates (*R* > 0.99) (Fig. S18B). Through a comprehensive methodology centered on global metabolomic profiling, unique metabolic patterns distinguishing leaf tissues from root tissues were identified. Numerous compounds exhibited notable changes (>2-fold; *P* < 0.05), specifically 423 compounds that were upregulated and 260 that were downregulated, identified as differential accumulation metabolites (DAMs) (Fig. S19A).

Among these DAMs related to KEGG, terpenoid biosynthesis was observed (Fig. S19B). Over 15.42% of the DAMs were associated with iridoids (Fig. S19B). Iridoids are important monoterpene analog in *S. ningpoensis* [56], synthesized from dimethyl ally diphosphate via the conserved mevalonate pathway (MVA) and methylerythritol 4-phosphate pathway (MEP) pathways in plants [57–59]. Genome annotation identified all candidate structural enzyme-coding genes involved in these pathways (Table S20; Fig. S20). Gene expression indicated active transcription in MVA pathway PCGs in roots and flowers, while the MEP pathway protein coding genes were high transcription activities in leaves (Fig. S21). These data were consistent with previous results observed in other medical plants [59]. It has been reported that enzymatic genes from the MEP pathway are chloroplast-localized but encoded by nuclear genes [60]. To probe the metabolic actions of H3K36me3 in iridoids biosynthesis in *S. ningpoensis*, H3K36me3-hypermethylated genes were mapped from DEGs and DAMs (Fig. 5G). As results, a ubiquitous transcriptional model in roots was checked for H3K36me3 methylated MVA genes, including AACT and PMK, while most MEP pathway genes were expressed in leaves (Fig. 5G).

### *SnSDG8*-directed H3K36me3 is essential for iridoids biosynthesis in *S. ningpoensis* under environmental warming

**Figure 6:**
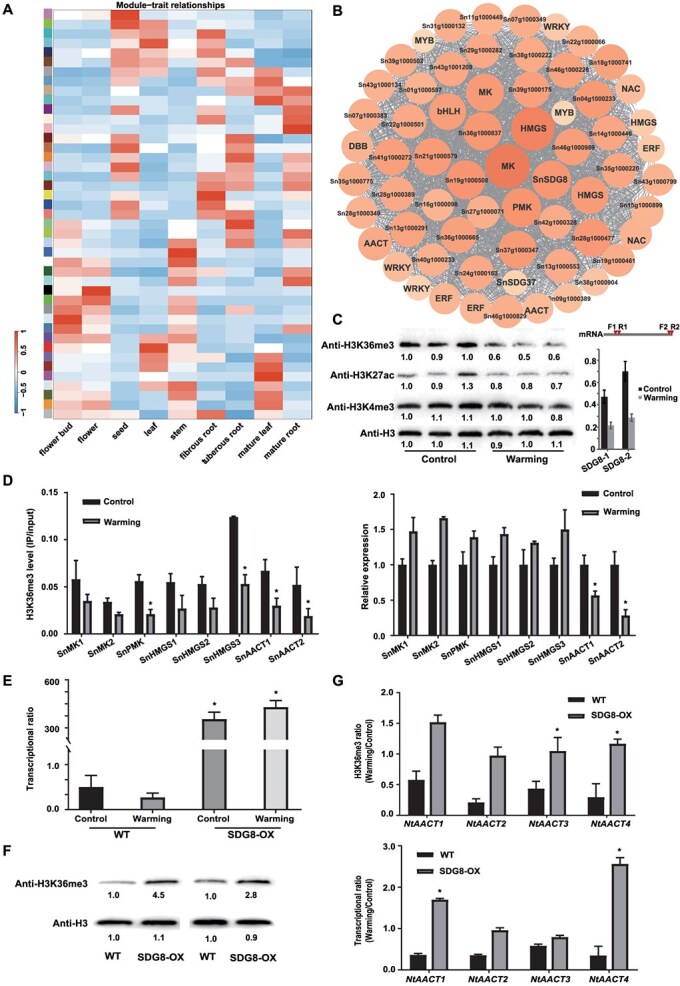
Functional identification of *SnSDG8* with the help of tobacco system. (**A**) The outcomes of the network analysis. Sample clustering for different tissues from WGCNA. **(B**) Differential expression genes clustered in a dendrogram are displayed alongside merged module colors and their original counterparts. Edge width represents connection strength (edges with weights <0.3 are not shown). (**C**) The total level of H3K36me3 in root tissues of *S. ningpoensis* in control and warming groups. H3 was as loading control. The histone signatures’ band was normalized with control with the ImageJ (1.54). (**D**) H3K36me3 levels of candidate genes. ChIP-qPCR (left) and expression (right) analysis was performed in samples. * *P* < 0.05 with Student *t*-test. (**E**) QR-qPCR assay for the expression levels of *SnSDG8* (SET domain group 8) in SDG8-overexpressed tobacco plants. (**F)**  *SnSDG8* is responsive for histone H3K36me3. (**G**) SnSDG8 regulates the transcribed levels of genes essential for iridoids biosynthesis genes in MVA pathway under warming stress. * *P* < 0.05 with *t*-test.

To probe the co-expression models related to the pathway, it was conducted an analysis of differential transcription and developed weighted gene co-expression networks analysis (WGCNA) with DEGs, resulting 43 clusters (Fig. 6A). Notably, key gene copies involved in iridoid biosynthesis were identified in the ‘blue’ module (*r* = 0.7, *P* = 4e-5) by WGCNA; they included *Sn27g1000020* and *Sn19g1000020* (encoding AACT), *Sn28g1000389* and *Sn32g1001015* (encoding HMGS), *Sn15g1000283* and *Sn21g1000251* (encoding MK), and *Sn23g1000987* (encoding PMK) (Fig. 6B). In *Arabidopsis*, the histone methyltransferase encoding gene *AtSDG8* was reported to regulate H3K36me3 deposition [24]. Notably, it was found that the homologues gene Sn18g1001580 (encoding *SnSDG8*) in *S. ningpoensis* was co-expressed with iridoid biosynthesis genes in the same module (Fig. 6B). In consistence, *SnSDG8* shared similar expression pattern with MVA pathway genes (Fig. S22), supporting the hypothesis that H3K36me3 preferentially marks iridoid biosynthesis genes in the MVA pathway (Fig. 5G) to regulate iridoids production in *S. ningpoensis*.

Given the enhancement of iridoids biosynthesis under environmental warming from this study (Fig. 1) and the requirement of *AtSDG8*-directed H3K36me3 in warming-induced flowering by previous study [23], it was performed a western blot to detect total H3K36me3 levels in controls and warming *S. ningpoensis* groups in root tissues (Fig. 6C). H3K36me3 levels significantly decreased under warming conditions, unlike H3K27ac and H3K4me3 marks, which also dominated in ASE genes (Fig. 4C). And meanwhile, it was also detected that the expression of SnSDG8 displayed much lower in warming group than those in control group (Fig. 4C). Additionally, the ChIP-qPCR assay found H3K36me3 levels were decreased within iridoid biosynthesis genes in MVA pathway under warming (Fig. 6D; Table S21). Interestingly, *SnAACT* (Acetoacetyl CoA thiolase) genes, which catalyzed the initial of MVA pathway [61], display down-regulated, exception for other related genes, which was the first biosynthesis enzyme in MVA pathway (Fig. 6D). Next, to confirm SnSDG8 as an active histone methyltransferase, it was performed *in vivo* enzymatic activity assays with the help of *Nicotiana benthamiana* system. As results, overexpression of *SnSDG8*-GFP (Fig. 6E) resulted in a marked increase in H3K36me3 compared to controls (Fig. 6F). Accordingly, to further investigation into *SnSDG8* involvement in iridoids synthesis, it was employed an exogenous *SnSDG8* overexpressing in tobacco leaf system. As results, it showed that *SnSDG8* indeed could enhance H3K36me3 levels in *NtAACT*, finally activating these rate-limiting biosynthetic genes in iridoid pathway (Fig. 6G). Overall, our findings suggested that H3K36me3-directed metabolic production can serve as markers in the response to changes of environmental warming during suitable cultivation of *S. ningpoensis*.

## Discussion

Not all temperature increases have negatively effects on plants [62]. Different cellular components may monitor temperature changes. While mild warmth induces thermomorphogenesis, excessive heat triggers acclimation responses that can hinder plant growth and development [63]. In line with previous studies on *S. ningpoensis* [26], our findings confirm temperature has a stronger correlation with iridoids production than other environmental factors (Fig. 1B). It was observed that moderate warming enhances iridoids biosynthesis, whereas excessive heat causes a decline in *S. ningpoensis* (Fig. 1C and D). Alternatively, despite a potential reduction in adaptive areas due to future climate changes, the quality and efficacy of cultivated *S. ningpoensis* may improve with global warming.

Understanding the epigenetic mechanisms of herbs respond to increasing temperatures aids in understanding the geoherbalism or ‘Daodi’ of TCM and breeding warming-acclimated medicinal plants [62]. Given that *S. ningpoensis* was sensitivity to surrounding temperature, it served as an ideal model for studying the herb geoherbalism, particularly its adaptability to increased temperatures [26]. To investigate the adaptability of *S. ningpoensis* to environmental temperature, it was first performed a high-quality chromone-level reference genome in chromosomal level (Fig. 1A; Table S2; Table S5; Table S6) and collected reliable epigenomic and metabolomic data from leaves and roots (Fig. S10; Fig. S18; Table S15). Additionally, transcriptome analysis was also conducted across nine developmental tissues (Table S13; Table S14). These multiomics datasets provide valuable insights into the environmental adaptation of *S. ningpoensis*. We reported a diploid genome with relatively high heterozygosity (Table S2), making *S. ningpoensis* a model to investigate allelic variations. This heterozygosity is likely tied to its outcrossing nature and self-incompatibility, similar to tea plants [64]. From allelic imbalance between two haplotypes, 3401 allele-specific expressed genes were identified, combined with transcriptomes from all developmental stages (Fig. 2; Table S13). These genes showed functional enrichment in response to stimulus pathways, which were not found in genes associated SVs (Figs S8 and S9). In plants, accumulating evidence suggest these genes are linked to temperature resistance [39,40]. Thus, allelic imbalance may contribute to the mechanism of warming response in herbs.

Trans-acting factors can influence both alleles within the nucleus, leading to ASE influenced by cis-acting polymorphisms, cis-acting epigenetic effects, or a combination of genetic and epigenetic factors [65]. CSs, characterized by various histone modifications and DNA methylations (Fig. 4B), showed enrichment of H3K27ac, H3K4me3, and H3K36me3 on the dominant side of ASEGs expression (Fig. 4C and D). Interestingly, DNA methylations seemed not contribute to this process (Fig. S13), in contrast to findings observed in the tomato genome [52]. This suggests that imbalanced transcriptional activities between the HA and HB genomes in *S. ningpoensis* may arise from distinct epigenetic statuses, which are not evolutionarily conserved in plant species. Additionally, the functional roles of H3K36me3 in modulating homologous gene pairs and haplotype-unique genes differed between roots and leaves (Fig. 5). H3K36me3 occupancy signals in ASEGs increased significantly (HA > HB), vice versa (Fig. 5A and B).

Central metabolic products act as substrates and cofactors essential for chromatin status; therefore, changes in metabolic fluxes can significantly impact the epigenome [66]. The interplay between metabolism and chromatin markers in plants continuously reshapes the epigenome and transcriptional activity, affecting plant development and their responses to environmental challenges [67]. Thus, combined with metabolomic data, H3K36me3 may act in a genic position dependent mode to reshape metabolic flux by promoting gene expression in the *S. ningpoensis* genome (Fig. 5G). From *S. ningpoensis*, 162 compounds have been isolated and identified, including iridoids, iridoid glycosides, phenylpropanoids, organic acids, essential oils, terpenes, sugars, flavonoids, sterols, and saponins. Among them, Iridoids, known for their bioactive properties, are key outputs of the widely conserved MVA and MEP pathways in plants [57–59]. The therapeutic efficacy of *S. ningpoensis* is primarily attributed to its iridoid glycosides and phenylpropanoid glycosides [56]. Most MVA associated genes actively transcribed in roots and flowers, predominantly marked by H3K36me3; while MEP pathway genes were evolutionarily detected in leaves (Fig. S21). Notably, H3K36me3 levels significantly decreased under warming (Fig. 6C), unlike H3K27ac and H3K4me3 marks, which also dominated in ASE genes (Fig. 4C). WGCNA, indicated that *SnSDG8*, the homolog of *AtSDG8* responsible for H3K36me3 deposition [24], was co-expressed with iridoids biosynthesis genes (Fig. 6B), sharing a similar expression pattern with MVA biosynthesis genes (Fig. S22). It was confirmed that SnSDG8 may reshape biosynthesis of iridoids under warming (Fig. 6F and G). In model plants, H3K36me3 plays a role in temperature-dependent alternative splicing under warming [23], potentially modulated through SDG8 [24].

## Conclusions

*Scrophularia ningpoensis* Hemsl., a TCM, has its bioactive compounds influenced by environmental temperatures during growth, making it an ideal model for studying the biological basis of TCM geoherbalism. However, the adaptive potential of epigenetic marks in *S. ningpoensis* under varying temperatures has been inadequately studied, partly due to lack of a reference genome. Herein, it was demonstrated that mild warm temperatures contribute to the metabolic accumulation and the cultivated migration of *S. ningpoensis* using a comprehensive global dataset. It assembled a nearly perfect genome at chromatin level, which generated an epigenetic atlas, metabolic, and transcriptomic profiles across different tissues. Transcriptome data from nine tissues revealed 3401 allele-specific expressed genes by comparing two haplotypes. Understanding how medicinal plants adapt to global warming is crucial, involving epigenetic mechanisms that alter phenotypes without changing DNA sequences. Using ChIP-seq and BS-seq data in leaf and root tissues, it was showed that ASEGs may be linked to distinct epigenetic patterns, particularly the active mark of H3K36me3, which appears to play varying functional roles in leaf and root tissues. Notably, genes marked with H3K36me3 from the iridoid synthesis pathway were ubiquitously transcribed in roots. Additionally, the histone methyltransferase SnSDG8 was identified to regulate ectopic H3K36me3 in iridoid biosynthesis in response to warming temperatures. Our results underscore the significance of epigenetic mechanisms in the context of global warming on herb-derived products, which is essential for medicinal plant breeding under temperature stress.

## Materials and methods

### Plant materials

Leaves of *S. ningpoensis* gathered at a plantation in Enshi, Hubei Province, China. Newly harvested of *S. ningpoensis* leaves underwent DNA extraction and sequencing. Total RNA was obtained from samples of *S. ningpoensis* flower buds, flowers, stems, leaves, seeds, fibrous roots, and tuberous roots at the full-blossom stage. Additionally, leaves and roots at a mature stage were utilized for the TRIzol reagent (Invitrogen) extraction process per the manufacturer’s guidelines. Leaf and root tissues collected at a mature stage were earmarked for BS-seq, ChIP-seq, and metabolome.

### Karyotype analysis

Flow cytometry analysis was according to a previously published procedure with slight modification [68]. Fresh and actively growing root tips from *S. ningpoensis* were placed into ice water with 24 h to gather metaphases before next step. Enzyme treatment involving cellulose and pectolyase digested the root tips, which were then squashed with acetic acid. Chromosomes were stained with DAPI. Imaging was performed with fluorescence microscope. Images were subsequently approach from Olympus Corporation. Designed probes targeting 5S ribosomal DNA or 18S rDNA were employed in fluorescence *in situ* hybridization to analyze the chromosomal ploidy characteristics of the samples.

### Illumina short-read sequencing

According our previous protocol [48], the leaf DNA was extracted using the DNeasy plant mini kit (Qiagen). Fragmentation of DNA was conducted in order to achieve DNA fragment sizes of approximately 350 base pairs. Subsequent to this, libraries for sequencing were prepared in accordance with the procedures described in the Nextera XT DNA Library Preparation Kit. These procedures encompassed repair of terminals, tail-A addition, integration of sequence adapter, purification of DNA, and amplification through PCR. Following this, an initial quantification step was carried out employing a 2.0 Fluorometer, and subsequent to this, each library was diluted to a concentration of 1 ng/μl. The length of the insert in each library was confirmed by means of an Agilent device. The Illumina DNA libraries were then subjected to sequencing on an Illumina platform.

### Genome survey

In order to evaluate genome size, as well as heterozygosity and repeat content, K-mer analysis was conducted utilizing Illumina DNA sequencing data with Jellyfish (v.2.1.4) [69]. The K-mers were tallied and combined, and histograms were output via the command of ‘-histo’. Using Genome Scope v.2.0, the histograms helped in determining both the genome size and heterozygosity [70].

### PacBio library construction and sequencing

After high quality of gDNA was extracted from leaves tissues, the gDNA was fragmented and subsequently collected employing magnetic beads. Fragment length was determined using an Agilent Bioanalyzer, and concentration was measured with a Qubit fluorometer to confirm sufficient DNA quantity for sequencing. Once the sample has passed the quality inspection, the library construction process begins with shearing the genomic DNA using gTubes. Following this, nuclease Exo VII was employed to digest and remove the single-stranded overhangs at the 3′ ends. A DNA damage repair kit was then used to address single-strand breaks, base deletions, oxidation, and other damages on the DNA strands. The DNA was further repaired to create blunt ends, and SMRT bell-shaped adapters were ligated. Nuclease digestion was performed again to eliminate any fragments that have not been ligated with the SMRT adapters. The library is then subjected to a secondary purification using 0.45X PB magnetic beads to yield the sequencing library. After the library construction was complete, its quality was assessed. Only when the library meets the required specifications, as determined by accurate quantification with a Qubit 3.0 and size verification with an Agilent 2100, was it deemed suitable for sequencing. Once the library passes these quality checks, it was ready to be sequenced using the PacBio Sequel ﻿II platform.

### Hi-C library building and sequencing

The methodology of preparing the Hi-C libraries involved freshly collected shoots and following the procedure outlined in a previously published study [71]. Initially, genomic DNA underwent cross-linking using formaldehyde, followed by nuclei extraction. Subsequently, the nuclei were digested using the MboI restriction endonuclease. The resulting fragments had their sticky ends biotinylated, followed by random dilution and ligation. To construct a total of nine sequencing libraries, the biotinylated DNA fragments were enriched.

### *De novo* genome assembly

Next, it was assembled *S. ningpoensis* genome by combining the sequences generated through HiFi reads and Hi-C technology using default parameters with Hifiasm [72]. Quality control measures were applied to the Hi-C reads using Juicer [73]. The contig assembly was then organized to chromatin scaffold using 3d-dna. The visualization of Hi-C interactions was analyzed using the 3d-dna and examined through Juicebox [74]. To evaluate the genome integrity at the chromatin level, BUSCO v.5.4.3 [75] (dataset: eudicots_odb10) was employed.

### Gene annotation

Repeats in *S. ningpoensis* genome were detected with an integration of ab initio and homology dependent prediction methods. For prediction based on homology, RepeatMasker and RepeatProteinMask from RepeatMasker v.4.0.7 [76] were utilized to detect repeats at both DNA and protein by searching against the RepBase library [77] and the TE protein database. Tandem repeats were characterized with Tandem Repeat Finder [78]. LTR_FINDER [79] and RepeatModeler [80] were *de novo* predicted of novel repetitive elements. To identify rRNA sequences, a BLASTN search was carried out against rRNA sequences of closely related species, with the alignment results used for annotation within the genome. The prediction of miRNA and snRNA was conducted using Rfam (v.1.0.4) [81]. tRNA sequences were obtained through the genome using tRNAscan-SE 1.3.1 software [82].

The prediction of PCGs in *S. ningpoensis* utilized a comprehensive approach combining *de novo* prediction and other prediction. Initially, the RNA-seq fragments underwent cleaning and alignment with *S. ningpoensis* genome using HISAT2 v.2.2.1 [83]. Subsequently, StringTie v.2.1.6 [84] identified potential exon regions, and ORFs were predicted via TransDecoder v.5.1.0 using the transcript sequences. Homologous protein sequences of *Abrus precatorius*, *Aegilops tauschii*, *Erythranthe guttata*, *Olea europaea*, and *Sesamum indicum* obtained from NCBI were aligned with the *S. ningpoensis* genome with TBLASTN. Exonerate﻿ v.2.2.0 [85] was employed to predict the precise CDS of each blast hit within the corresponding genomic region. Additionally, it predicted the *de novo* gene structure with AUGUSTUS v.3.2.3 [86] and GlimmerHMM v.3.0.4 [87] on the repeat database. Integration of all gene structure results from ab initio, homology, and transcriptome predictions with MAKER software (v.2.31.8) [88] resulted in a set of high-quality protein-coding genes for *S. ningpoensis*. It functionally annotated the predicted PCGs was performed with the help of various publicly available databases such as Swissprot [89], TrEMBL (**http://www.uniprot.org/**), InterPro [90], GO, KEGG [91], and NR (http://www.ncbi.nlm.nih.gov/protein/) databases.

### Gene family characterization and phylogeny analysis

Gene family was identified between *S. ningpoensis* and other 12 species (*Buddleja alternifolia*, *Strobilanthes cusia*, *S. indicum*, *Jacaranda mimosifolia*, *Boa hygrometrica*, *Syringa oblata*, *Gardenia asminoides*, *Morinda officinalis*, *Catharanthus roseus*, *Camptotheca acuminate*, *Arabidopsis thaliana*, and *Vitis vinifera*) using Orthofinder2 [92]. Each of the gene collections from the 13 species was refined based on a criterion where only the longest transcript was kept in cases of numerous alternative splicing transcripts. Protein sequence similarity was evaluated through BLASTP. In total, 191 single-copy orthologous genes were characterized. Execution of protein alignments for each orthogroup was done by MAFFT [93]. These alignments were refined by eliminating sites with more than 50% gaps and excluding sequences that were shorter than 50% of the alignment's length. To construct the species tree, all 381 loci were utilized for analysis using IQ-Tree [94]. To evaluate branch supports, ultrafast bootstrap and SH-aLRT analyses were conducted with 1000 replications.

According to outcome of the phylogenetic tree, the estimation of species divergence time could be carried out with the mcmctree tool in PAML (v.4.9) [95]. The calibration reference for the divergence examination was extracted from the TimeTree online platform. The visualization of the phylogenetic tree included a 95% highest posterior density interval. To analyze gene family contractions and expansions, the CAFE5 [96] software was employed, utilizing the gene family clustering data. CAFE5 introduced a stochastic model of birth and death to calculate the λ value.

### Identification of WGD in *S. ningpoensis*

In order to acquire syntenic blocks, protein sequences were aligned among *S. ningpoensis*, *B. alternifolia*, *S. indicum*, and *V. vinifera* using BLASTP. Syntenic section displaying collinearity among paralog pairs were characterized via MCscanX [37]. The Ks-based values for all paralogous genes within these genomes of the *S. ningpoensis*, *B. alternifolia*, *S. indicum*, and *V. vinifera* genomes was established. Each paralogous pair was aligned using MAFFT, and Ks estimation for all pairwise comparisons within a paralog pair was done using the CODEML program in PAML. ParaAT (v.2.0) [97] was utilized to generate numerous PCGs sequences, which acted as input values for KaKs_Calculator [98] to compute the Ks values. The 4-fold degenerate synonymous site (4dTv) of the third codon in syntenic segments was determined using conjunctional alignments. Following this, the 4dTv values were visually depicted to show their distribution. The peak values in this distribution were then used to infer WGD event.

### Identification of alleles

To detect homologous sequences between HA and HB, it was utilized the MCscanX [37] for constructing the syntenic blocks. Our analysis of syntenic sequences adhered to specific guidelines: (i) paired regions must be located on matching haplotypes, (ii) the length of one segment was not permitted to exceed three times that of its counterpart, and (iii) the regions in alignment had to encompass a minimum of 50% of the entire area. Those regions that met these stipulations were recognized as syntenic regions. A gene and its closest homologous counterpart on the opposite haplotype were identified and termed as allelic genes.

### RNA sequencing and analysis

For one sample, 2 μg of RNA was utilized for RNA preparation. The mRNA isolated from total RNA using magnetic beads. Utilizing Illumina library Kit, library was produced according to the guidelines, and index were incorporated to map sequence. Subsequent sequencing was performed on an Illumina NovaSeq 6000 platform. To ensure data quality, RNA-seq data underwent initial cleaning to eliminate contaminants and bad reads using Fastp [99]. The clean data were mapped with the *S. ningpoensis* genome as the reference with HISAT2 [83]. Gene numbers were then determined with Rsubread (v.2.10.5) [100]. Identification of DEG was performed with DESeq2, followed by functional analysis of these genes with GO and KEGG through custom scripts.

### ASE

RNA-seq reads were obtained for nine different tissue types (flower buds, flowers, stems, leaves, seeds, fibrous roots and tuberous roots at full-blossom stage, and leaves and roots at mature stage) with three biological replicates. Normalized transcripts per million values were analyzed to identify variations in allele expression. Differential expression of alleles was determined based on FC values within the ranges of 8 ≥ FC ≥ 2 or FC > 8 or 2 > FC ≥ 0, while maintaining *P* value <.05.

### Gene co-expression network

Allelic expressions were chosen for constructing co-expression networks through the utilization of the WGCNA [101]. The original threshold of power was identified as the least amount of power needed to exceed an index of 0.85 for a topology fit for every network. Matrices for topographical overlap were calculated using the modules function in a blockwise manner, with TOMType = ‘unsigned’ and a minimum module size of 40 being established. To combine comparable modules, the CutHeight parameter is set to 0.15.

### ChIP-seq sequencing and analysis

ChIP experiments were performed as previous protocol [46]. Approximately 2 g underwent cross-linking with formaldehyde. And then, the chromatin was isolated and smeared into smaller fragments through sonication. Anti-H3K9ac (ab32129) was utilized for the ChIP procedure. The ChIP DNA was used to build libraries according to the Illumina protocol. Subsequent sequencing was conducted on the Illumina platform. ChIP-seq sequences were aligned to the *S. ningpoensis* genome with bowtie2 [102]. Alignment sorting and removal of potential PCR duplicates and low-quality alignments were carried out using samtools [103]. Deeptools [104] employed to generate a correlation plot for all data. The IP signal enrichments were used to generate a heatmap with the help of Deeptools. MACS3 software [105] was used to identify marked regions and call peaks, which were subsequently annotated using ChIPSeeker [106]. Differentiation was analyzed with Bedtools and DiffBind. BigWig format was generated with MACS3 that used for visualization by IGV [107]**.**

### BS-Seq analysis

The DNA was fragmented into 350 bp pieces, and blunt-ended fragments were produced. To ensure the fragments were suitable for ligation, phosphorylation of the 5′ ends was then performed. The repaired and phosphorylated fragments were dA-tailing at the 3′ end. Adaptors with methylated sites necessary for next steps were then ligated to the fragments with dA. The selection size was achieved within the 300–600 bp range through magnetic bead separation. The quantification of the WGBS was conducted with the Qubit 3.0, and the insert sizes were elevated with the Agilent device. The molality of the library was determined with the StepOnePlusTM Real-Time PCR system, with a requirement of more than 10 mM for sequencing. For sequencing the WGBS library, Illumina sequencers were used with 150-bp reads.

Evaluation of WGBS was conducted with FastQC software, followed by the removal of adapter and poor reads with fastp [94]. The clean reads were then aligned to the genome of *S. ningpoensis* utilizing Bismark [108]. The determination of the methylation status for each cytosine site was achieved by calculating the ratio of methylated reads over the site's total coverage, thus generating a methylation level ranging between 0 and 1 for each site. To evaluate the methylation level across a region, the aggregate count of methylated reads in that region was divided by the site's total coverage. Methylation profiles were developed utilizing 20 bins spanning the gene body and its adjacent regions (within a 1 kb range) and were created using an R script. This modification incorporates different phrasing, maintains the original meaning, and addresses potential duplication concerns.

### Identification of CSs and map of genomic features

In order to assess the importance of the integrated data related to various histone modifications, it was employed ChromHMM v.1.24 [109]. Read counts from each histone modification (H3K4me2, H3K4me3, H3K9me2, H3K27me2, H3K27me3, H3K36me2, H3K36me3, H3K9ac, and H3K27ac) were calculated within distinct 200-bp bins for the CS. A range of 10–20 states was evaluated, and a model with 15 states was chosen for further analysis.

### Metabolome profiling and statistical analysis

Leaf and root tissues at the mature stage were processed using a vacuum freeze-dryer until fully freeze-dried and subsequently ground into powders. For the preparation, lyophilized powders were measured and dissolved in 1.2 ml of 70% methanol. The resulting solutions were then subjected to vortex mixing, repeated six times, and followed by refrigeration overnight. The supernatants were filtered through membrane to collect the extracts, which were then analyzed using UPLC–MS/MS analysis. The metabolomics were executed on a UPLC–ESI–MS/MS system, managed by MetWare Biotechnology according to their established protocols. To determine obviously regulated metabolites, criteria of VIP values greater than or equal to 1 and an absolute log2FC (fold change) greater than or equal to 1 were employed. Analysis utilized MetaboAnalystR [110], extracting VIP values from the OPLS-DA result.

### Western blotting

According to our previous protocol with slight modification [111], histone fractions were extracted and enriched from *S. ningpoensis* roots and tobacco leaves using an EpiQuik Total Histone Extraction Kit (OP-0006-100; Epigentek) according to the manufacturers’ instructions. The histone-enriched fractions were used for immunoblot analysis. Antibodies used in the immunoblotting assay included anti-H3 (ab1791; Abcam), anti-H3K36me3 (ab9050; Abcam), anti-H3K27me3 (ab6002; Abcam), and anti-H3K4me3 (ab8580; Abcam). The band intensities were quantified using ImageJ software.

### Identification of iridoid biosynthetic pathway associated genes

Iridoids are synthesized through either the plastidial MEP pathway or the MVA pathway. By performing a KEGG annotation combined with a BLASTP search against the genome of *S. ningpoensis*, we pinpointed all relevant genes linked to the MEP and MVA pathways. This enabled us to map out the complete biosynthesis pathway for iridoids. Gene expression profiles were depicted using heatmaps, utilizing RNA-seq data as the foundation.

## Supplementary Material

Web_Material_uhae328

## Data Availability

The raw sequence data included in this manuscript have been deposited in the Genome Sequence Archive at the BIG Data Center of the Beijing Institute of Genomics (BIG), Chinese Academy of Sciences. Access to this data is available to the public under the accession number PRJCA026437, which can be found at the following URL: https://bigd.big.ac.cn/gsa/.
