# Supplementary material for: A chromosome-scale genome assembly and epigenomic profiling reveal temperature-dependent histone methylation in iridoid biosynthesis regulation in Scrophularia ningpoensis
Source: Hortic Res. 2025 Mar 4;12(3):uhae328. doi: 10.1093/hr/uhae328 (PMC11879554; doi:10.1093/hr/uhae328)
Supplement: Web_Material_uhae328 [file web_material_uhae328.zip › Supplemetary Figure10.pdf]

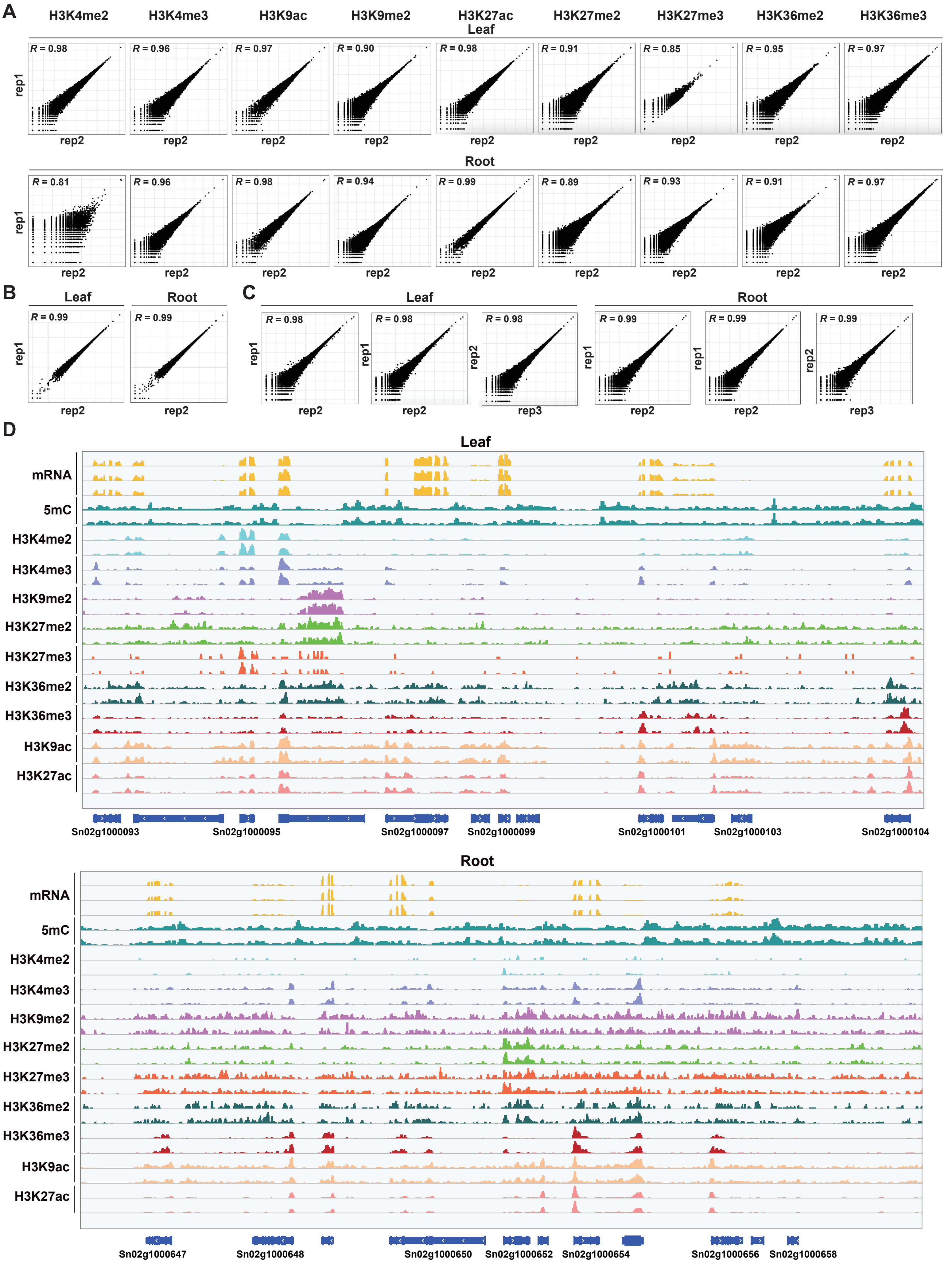

Fig. S10 Data reproducibility of epigenome data in this study.
(A-C) Scatterplots of enrichment scores compared across leaf and root of S. ningpoensis in biological triplicates of ChIP-seq (A), BS-seq (B) and RNA-seq (C). Pearson correlation coefficient values are shown at the top left corner of each plot. **(D)** Snapshots illustrating the enrichment of ChIP-seq, BS-seq and RNA-seq.
