# Supplementary material for: A chromosome-scale genome assembly and epigenomic profiling reveal temperature-dependent histone methylation in iridoid biosynthesis regulation in Scrophularia ningpoensis
Source: Hortic Res. 2025 Mar 4;12(3):uhae328. doi: 10.1093/hr/uhae328 (PMC11879554; doi:10.1093/hr/uhae328)
Supplement: Web_Material_uhae328 [file web_material_uhae328.zip › Supplemetary Figure11.pdf]

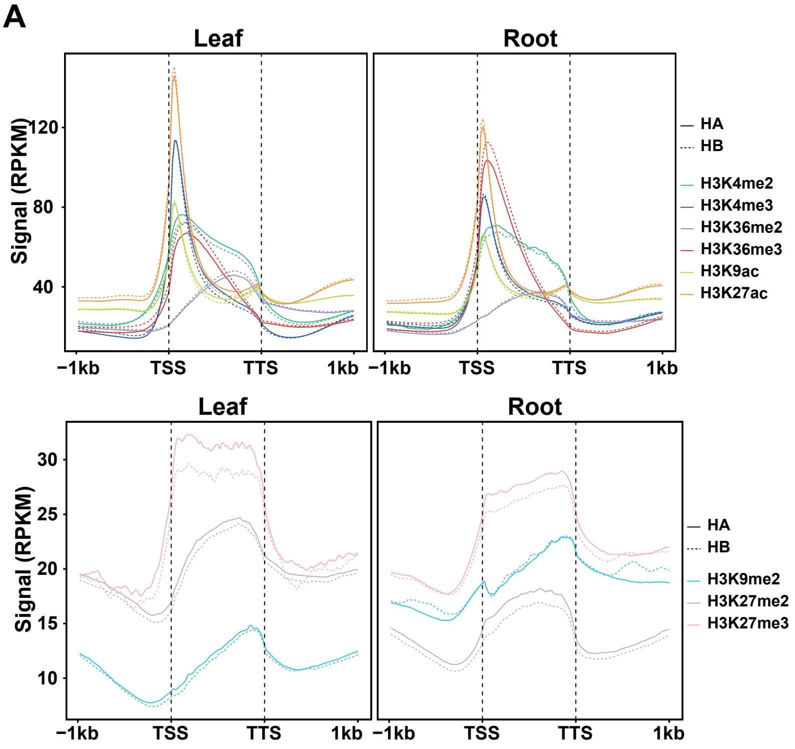

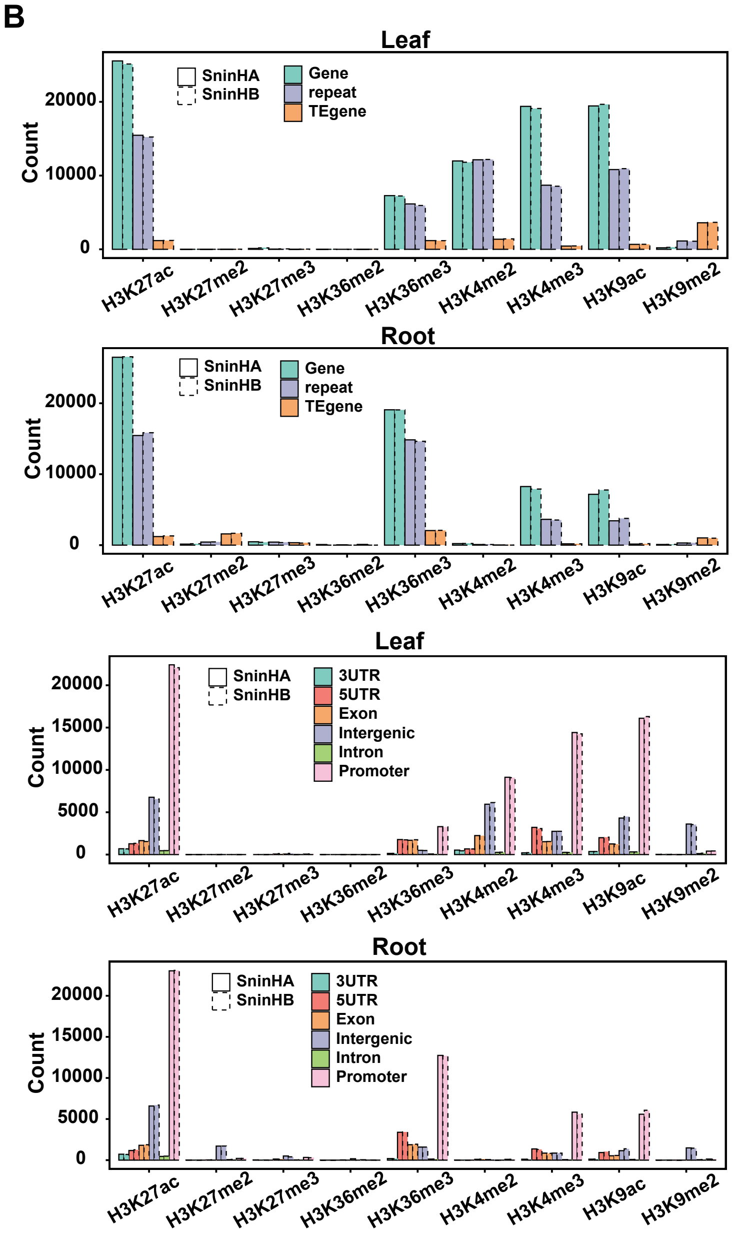

C

Tissues	Methylation Levels			
	C	CG	CHG	CHH
Leaf	25.15%	81.07%	54.23%	10.37%
Root	27.93%	81.25%	55.16%	14.01%

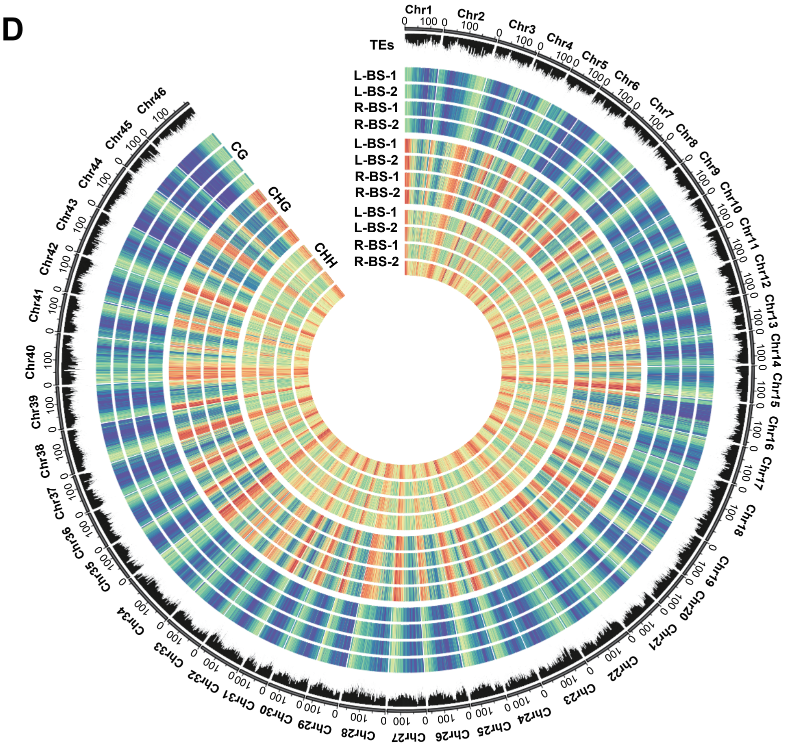

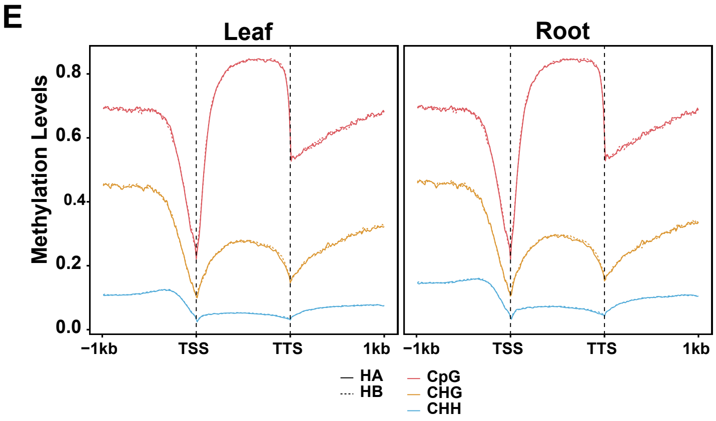

Fig. S11 Global distribution of epigenetic marks in *S. ningpoensis* genome.

(A) Distribution of histone marks occupancy along genes in leaf and root tissues of *S. ningpoensis*. **(B)** The counts of histone marked gene, repeat,TE gene, and genic and intergenic regions. **(C)** Methylation levels in leaf and root tissues of *S. ningpoensis*. **(D)** Global distribution of DNA methylation in the two tissues of *S. ningpoensis* genome. Methylation density is represented in 500kb blocks, separated by context and cell type. CG (CG methylation), CHG (CHG methylation). The outer annotation track highlights the position of transposons (TEs). **(E)** Distribution of DNA methylation in leaf and root tissues of *S. ningpoensis*.
