# Supplementary material for: A chromosome-scale genome assembly and epigenomic profiling reveal temperature-dependent histone methylation in iridoid biosynthesis regulation in Scrophularia ningpoensis
Source: Hortic Res. 2025 Mar 4;12(3):uhae328. doi: 10.1093/hr/uhae328 (PMC11879554; doi:10.1093/hr/uhae328)
Supplement: Web_Material_uhae328 [file web_material_uhae328.zip › Supplemetary Figure12.pdf]

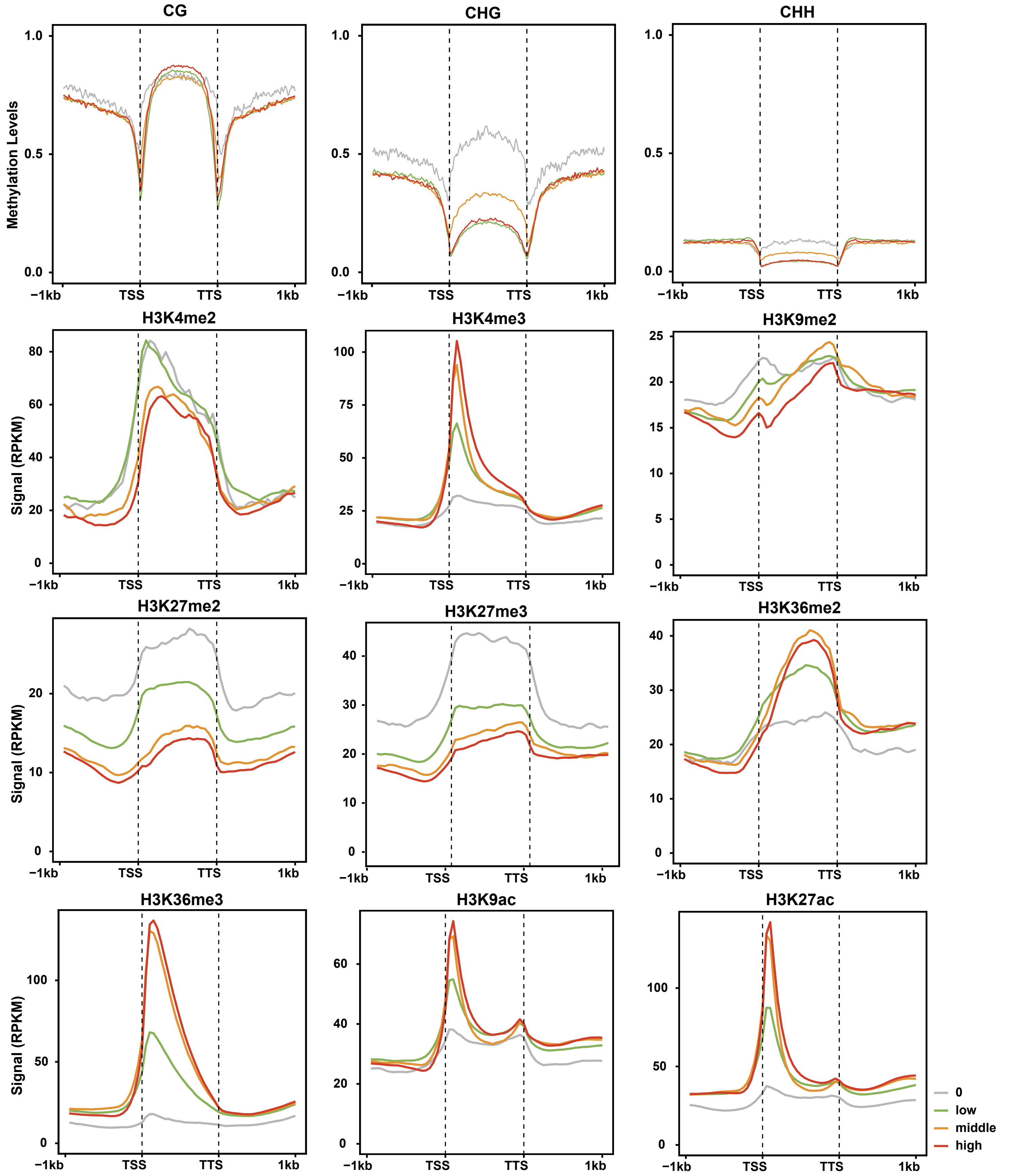


**Fig. S12 Epigenetic marks in relation to transcriptional activity in *S. ningpoensis* genome.**
Average genome-wide occupancies of multiple histone modifications and DNA methylation. Genes are classified according to their expression level.
