# Supplementary material for: A chromosome-scale genome assembly and epigenomic profiling reveal temperature-dependent histone methylation in iridoid biosynthesis regulation in Scrophularia ningpoensis
Source: Hortic Res. 2025 Mar 4;12(3):uhae328. doi: 10.1093/hr/uhae328 (PMC11879554; doi:10.1093/hr/uhae328)
Supplement: Web_Material_uhae328 [file web_material_uhae328.zip › Supplemetary Figure13.pdf]

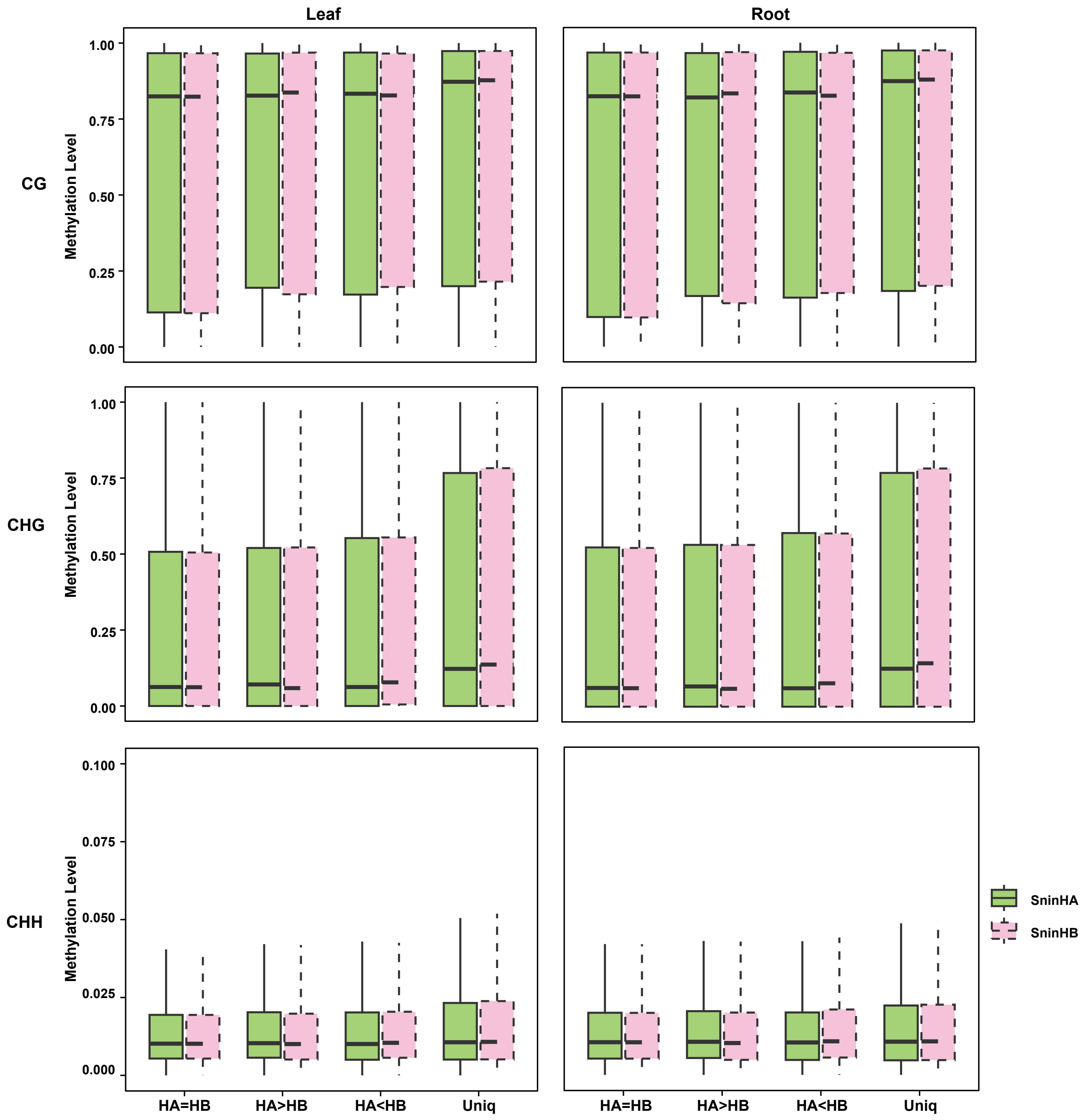

Fig. S13 Comparsion of DNA methylation in HA and HB chromosomes in leaf and root tissue of *S. ningpoensis.*
