# Supplementary material for: A chromosome-scale genome assembly and epigenomic profiling reveal temperature-dependent histone methylation in iridoid biosynthesis regulation in Scrophularia ningpoensis
Source: Hortic Res. 2025 Mar 4;12(3):uhae328. doi: 10.1093/hr/uhae328 (PMC11879554; doi:10.1093/hr/uhae328)
Supplement: Web_Material_uhae328 [file web_material_uhae328.zip › Supplemetary Figure14.pdf]

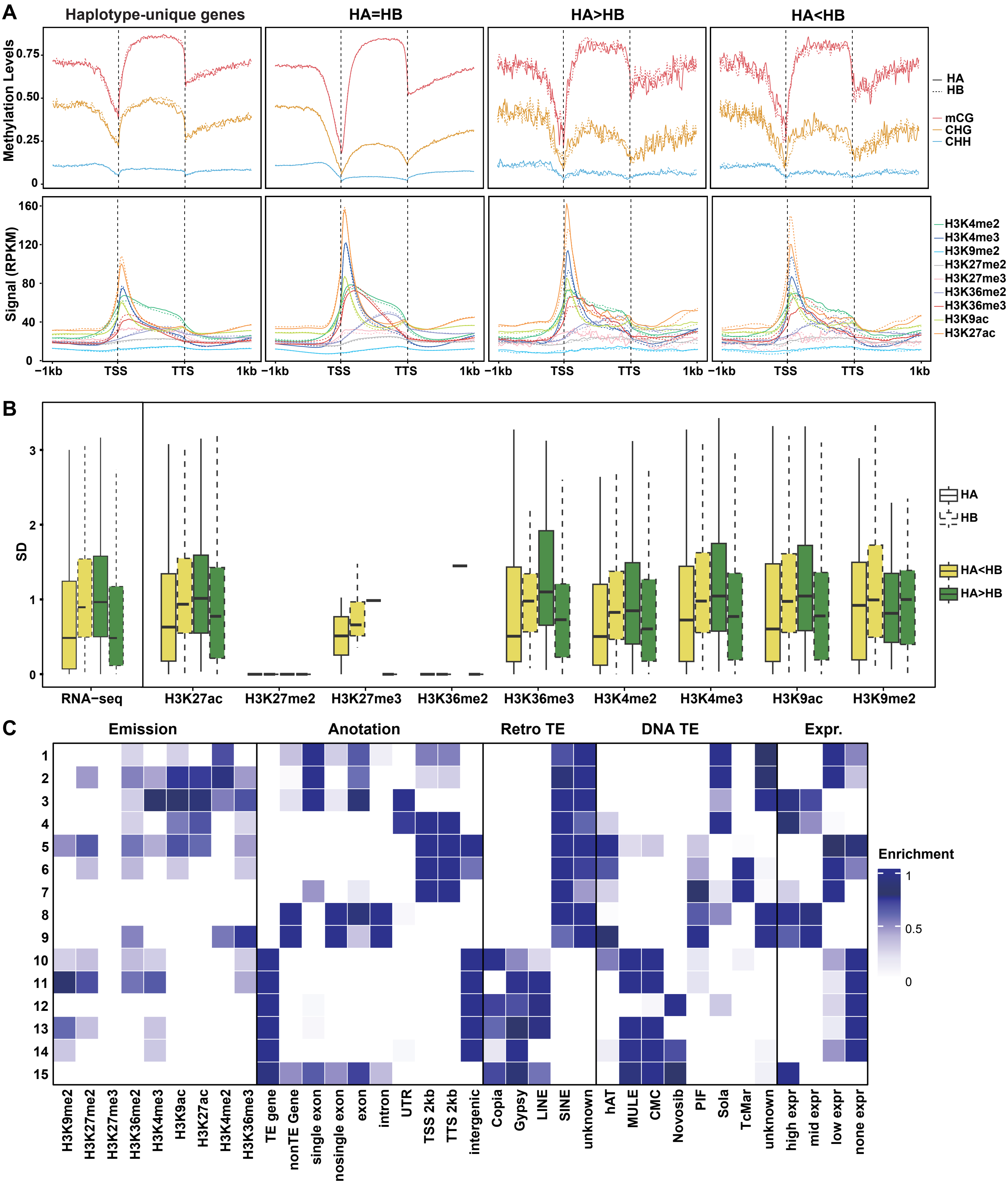

Fig. S14 Overview of chromatin states, DNA methylation and gene expression in HA and HB chromosomes from leaf tissue of *S. ningpoensis*.

(A) Epigenetic marks enrichment within transcriptional regions in the HA and HB genomes. **(B)** Variability of transcription, DNA methylation and histone modification occupancy of homeologous and subgenome-unique genes between the HA and HB chromosomes. **(C)** Enrichments for 15-state model based on five histone marks occupancy in the leaf tissue.
