# Supplementary material for: A chromosome-scale genome assembly and epigenomic profiling reveal temperature-dependent histone methylation in iridoid biosynthesis regulation in Scrophularia ningpoensis
Source: Hortic Res. 2025 Mar 4;12(3):uhae328. doi: 10.1093/hr/uhae328 (PMC11879554; doi:10.1093/hr/uhae328)
Supplement: Web_Material_uhae328 [file web_material_uhae328.zip › Supplemetary Figure16.pdf]

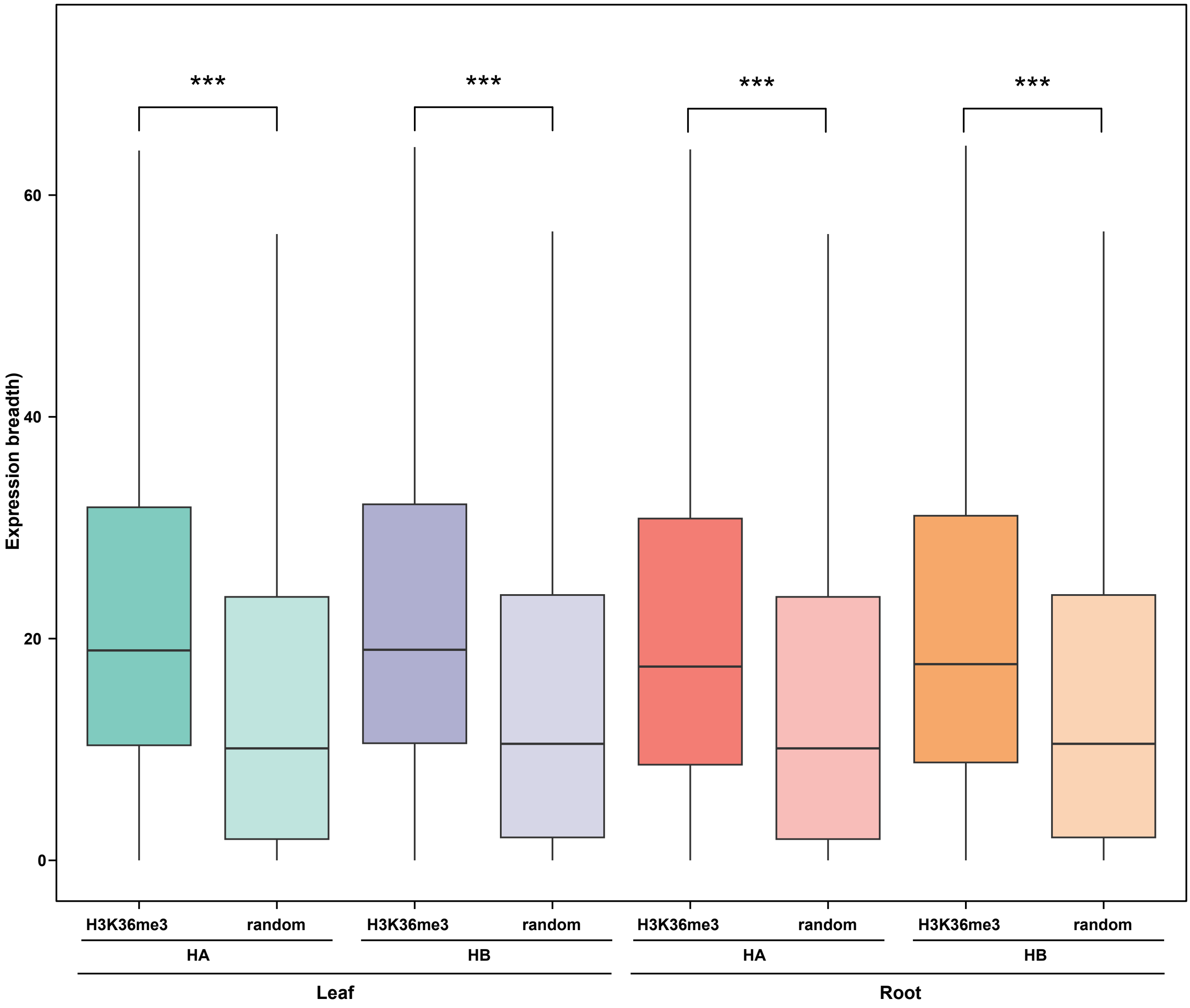

Fig. S16 Expression breadth of genes in the indicated categories.
The Wilcoxon rank-sum test was used for testing the statistical significance of differences (***$P < 0.001$); “random” refers to 10 000 randomly selected genes.
