# Supplementary material for: A chromosome-scale genome assembly and epigenomic profiling reveal temperature-dependent histone methylation in iridoid biosynthesis regulation in Scrophularia ningpoensis
Source: Hortic Res. 2025 Mar 4;12(3):uhae328. doi: 10.1093/hr/uhae328 (PMC11879554; doi:10.1093/hr/uhae328)
Supplement: Web_Material_uhae328 [file web_material_uhae328.zip › Supplemetary Figure19.pdf]

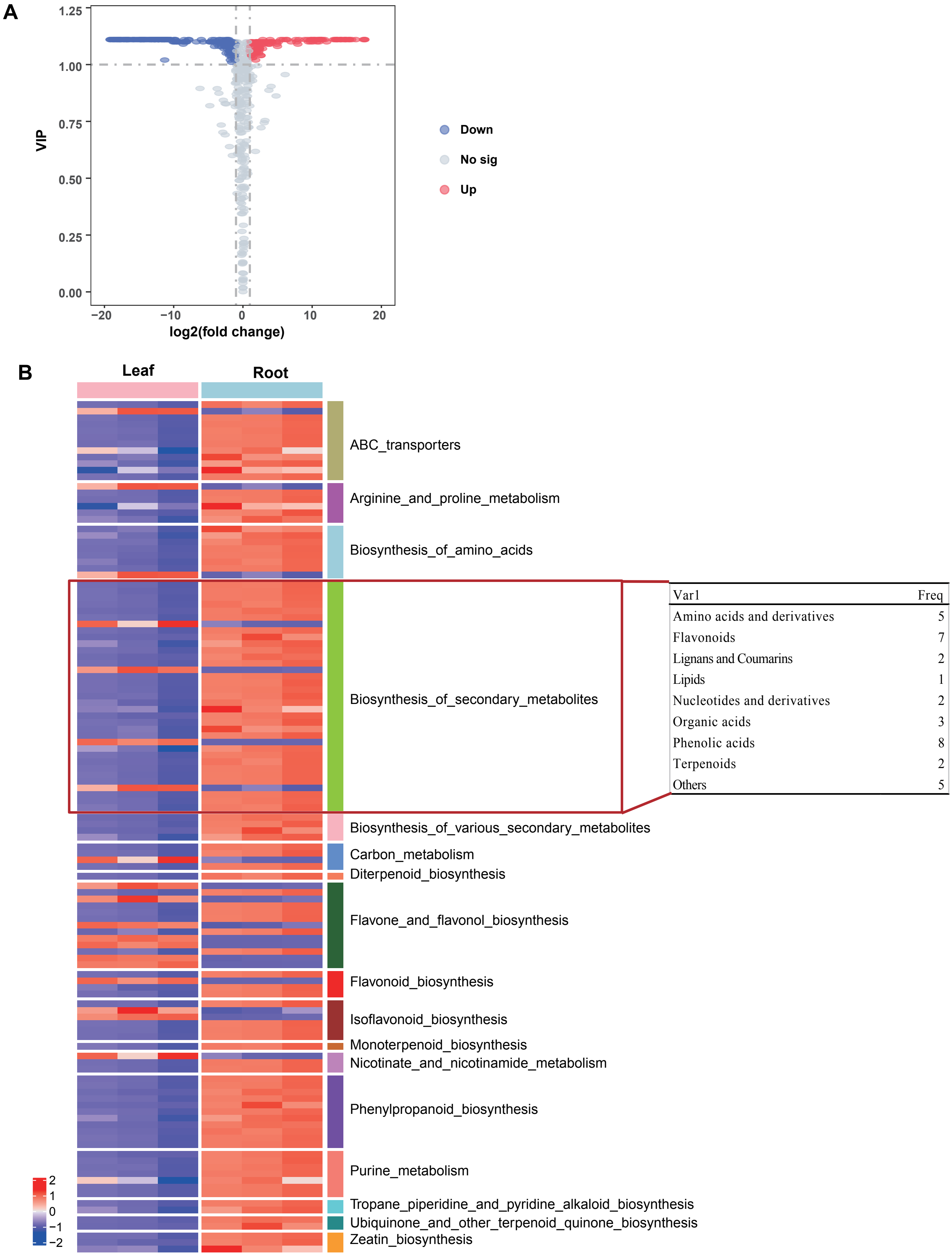

Var1	Freq
Amino acids and derivatives	5
Flavonoids	7
Lignans and Coumarins	2
Lipids	1
Nucleotides and derivatives	2
Organic acids	3
Phenolic acids	8
Terpenoids	2
Others	5

Fig.S19 The information of DAMs(differential accumulated metabolites) between leaf and root in *S. ningpoensis.*
(A) Volcanic map of differential metabolites. **(B)** KEGG enrichment map of differential metabolites.
