# Supplementary material for: A chromosome-scale genome assembly and epigenomic profiling reveal temperature-dependent histone methylation in iridoid biosynthesis regulation in Scrophularia ningpoensis
Source: Hortic Res. 2025 Mar 4;12(3):uhae328. doi: 10.1093/hr/uhae328 (PMC11879554; doi:10.1093/hr/uhae328)
Supplement: Web_Material_uhae328 [file web_material_uhae328.zip › Supplemetary Figure20.pdf]

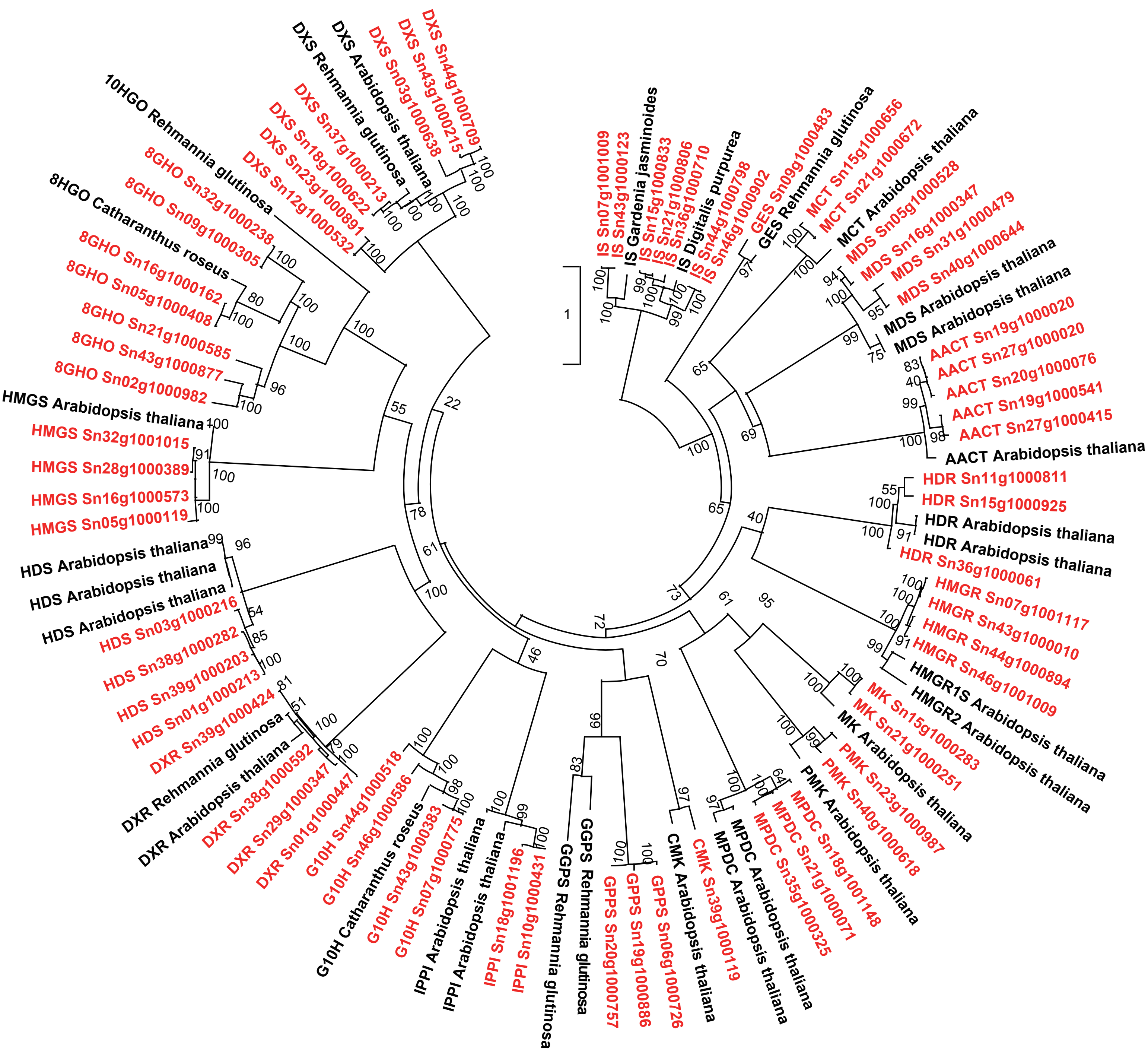

Fig. S20 A phylogenetic tree of all candidate iridoids biosynthesis gene families.
Numbers on the branches represent bootstrap support (%). The red represents the candidate iridoids biosynthesis genes of the *S. ningpoensis.*
