# Supplementary material for: A chromosome-scale genome assembly and epigenomic profiling reveal temperature-dependent histone methylation in iridoid biosynthesis regulation in Scrophularia ningpoensis
Source: Hortic Res. 2025 Mar 4;12(3):uhae328. doi: 10.1093/hr/uhae328 (PMC11879554; doi:10.1093/hr/uhae328)
Supplement: Web_Material_uhae328 [file web_material_uhae328.zip › Supplemetary Figure22.pdf]

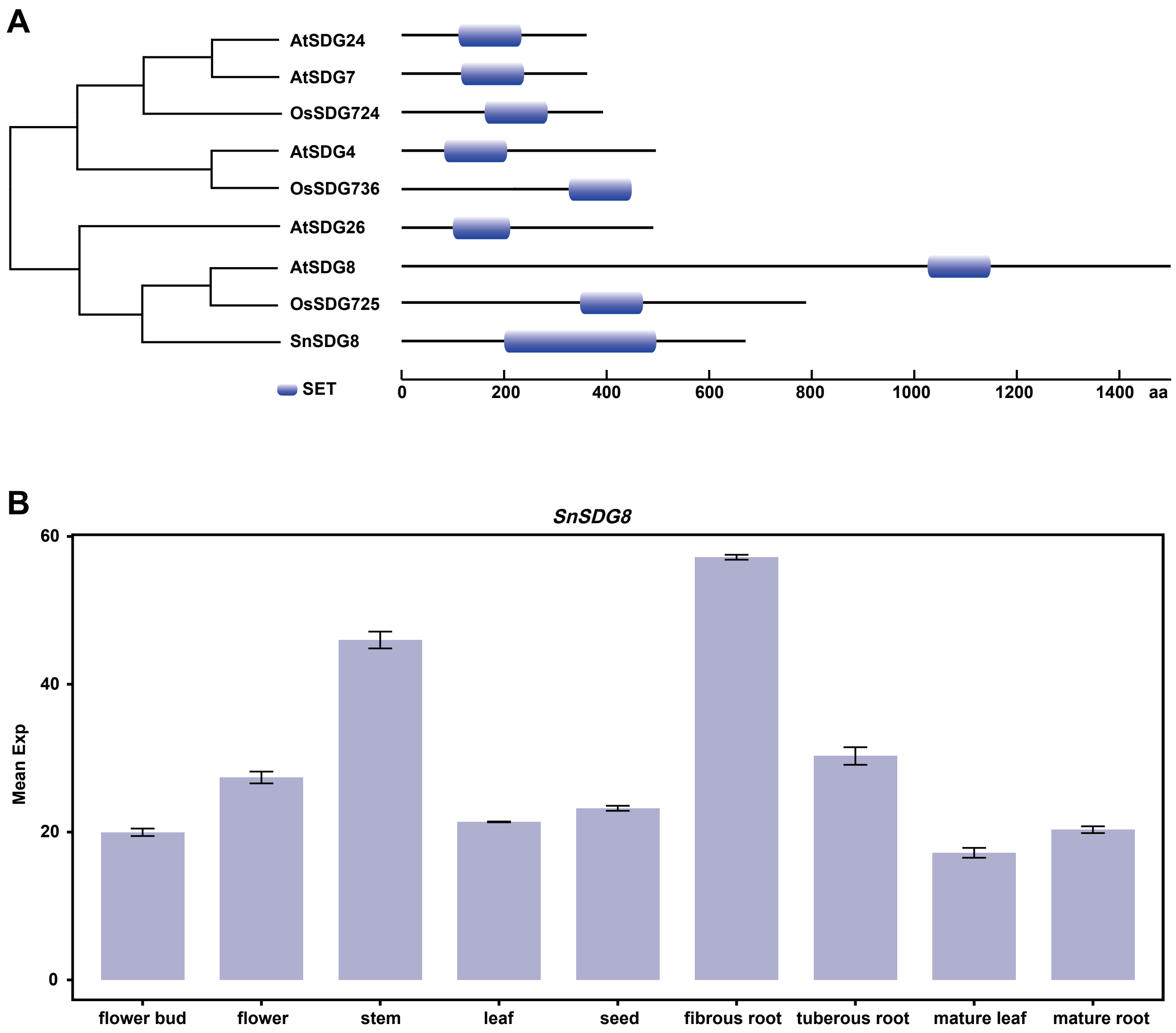

Fig. S22 The detailed information of *SnSDG8*.
(A) A phylogenetic tree of *SDG8* in *A. thaliana*, rice, tobacoo, *R. glutinosa*, *S. ningpoensis*. **(B)** Expression patterns of *SnSDG8* during the full developmental stages.
