# Supplementary material for: A chromosome-scale genome assembly and epigenomic profiling reveal temperature-dependent histone methylation in iridoid biosynthesis regulation in Scrophularia ningpoensis
Source: Hortic Res. 2025 Mar 4;12(3):uhae328. doi: 10.1093/hr/uhae328 (PMC11879554; doi:10.1093/hr/uhae328)
Supplement: Web_Material_uhae328 [file web_material_uhae328.zip › Supplemetary Figure3.pdf]

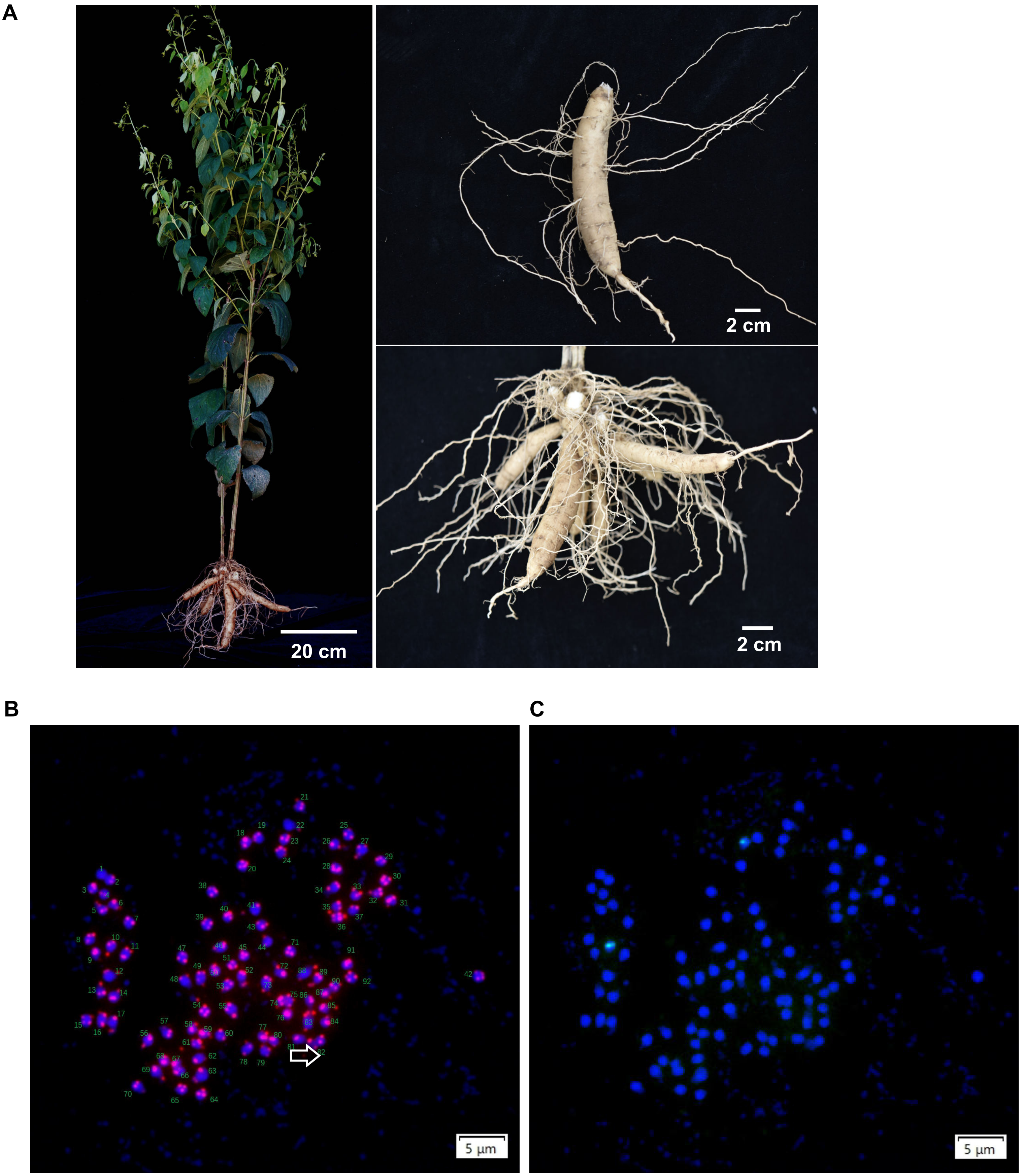

Fig. S3 Karyotype analysis.
(A) Image of whole plant and root tissue of *S. ningpoensis*. **(B)** Results of telomere chromosome fluorescence in situ hybridization in *S. ningpoensis*. Telomere fluorescence in situ hybridization signal was found at the end of chromosomes, which proved that the number of chromosomes was 92. The telomeres are red. **(C)** Results of 5S rDNA chromosomal fluorescence in situ hybridization of *S. ningpoensis* (arrows), indicating that the sequenced individual was diploid.
