# Supplementary material for: A chromosome-scale genome assembly and epigenomic profiling reveal temperature-dependent histone methylation in iridoid biosynthesis regulation in Scrophularia ningpoensis
Source: Hortic Res. 2025 Mar 4;12(3):uhae328. doi: 10.1093/hr/uhae328 (PMC11879554; doi:10.1093/hr/uhae328)
Supplement: Web_Material_uhae328 [file web_material_uhae328.zip › Supplemetary Figure4.pdf]

A

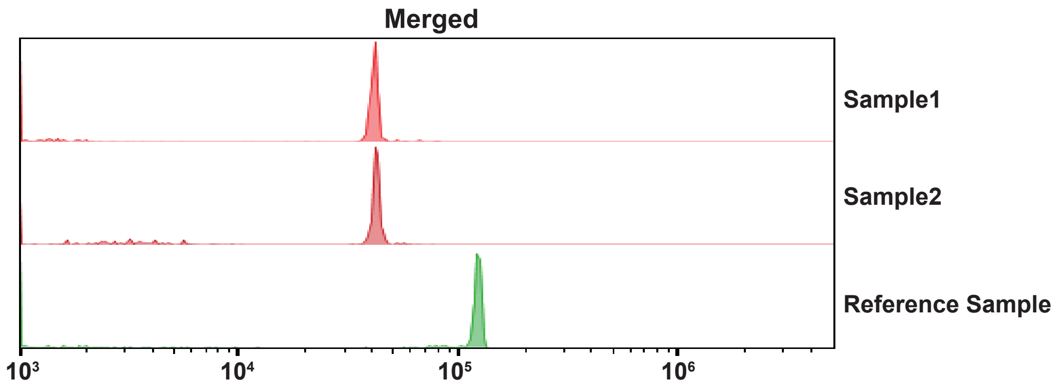

B

Statistical result

Species	Fluorescence mean value	Multiple	Genome Size (Gb)
Sample1	40,335.4	0.33	0.72
Sample2	40,715.1	0.34	0.73
Reference Sample	121,284.9	1.00	2.18

C

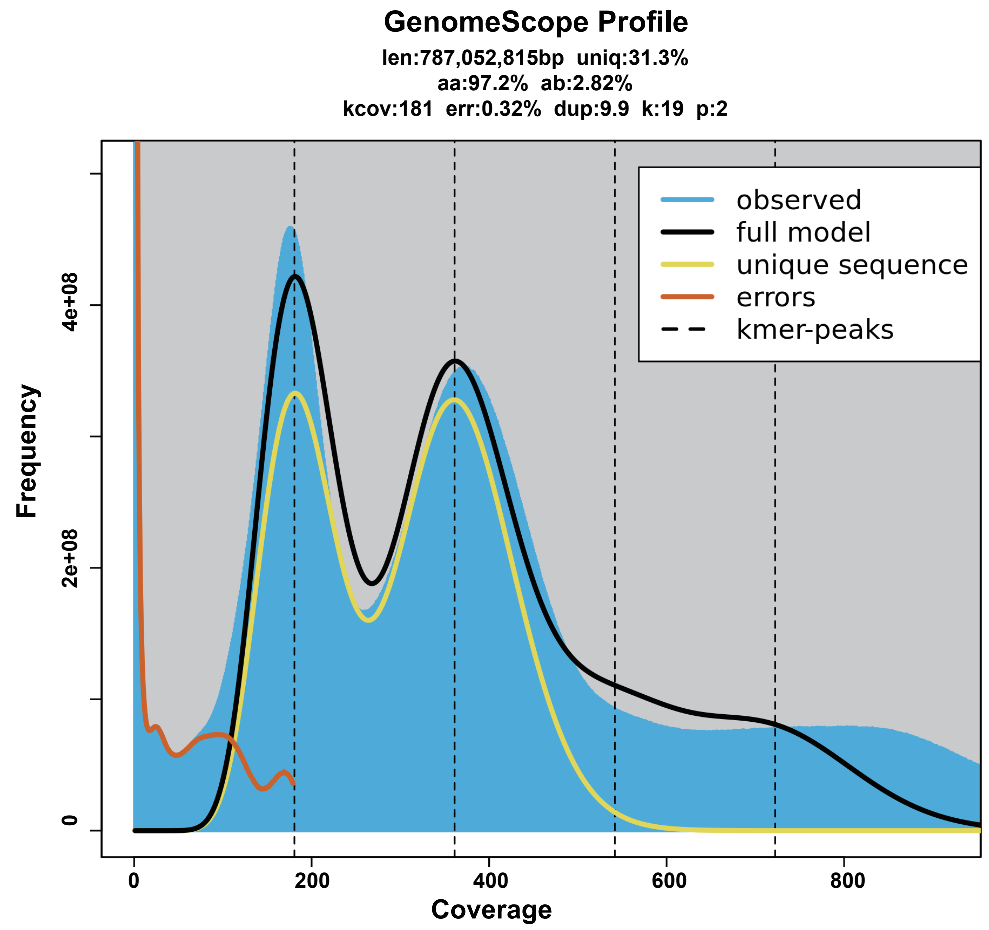

Fig. S4 Genome size estimation.
(A) Flow cytometry results of reference sample and *S. ningpoensis* samples 1 and 2. The characteristic peaks of each sample in G1 phase were shown in the form of histogram. The G1 phase characteristic peaks of *S. ningpoensis* samples 1 and 2 are shown in red, and the G1 phase characteristic peaks of reference sample are shown in green. Flowjo software was used to superposition the flow histograms of *S. ningpoensis* and reference sample to display the relative positions of characteristic peaks. **(B)** The mean fluorescence values of *S. ningpoensis* samples 1 and 2, reference sample, and the genome size of *S. ningpoensis* were calculated by external standard method. **(C)** K-mer distribution map of *S. ningpoensis* genome. The x-axis is the 19-mer depth, and the y-axis is the 19-mer frequency at that depth.
