# Supplementary material for: A chromosome-scale genome assembly and epigenomic profiling reveal temperature-dependent histone methylation in iridoid biosynthesis regulation in Scrophularia ningpoensis
Source: Hortic Res. 2025 Mar 4;12(3):uhae328. doi: 10.1093/hr/uhae328 (PMC11879554; doi:10.1093/hr/uhae328)
Supplement: Web_Material_uhae328 [file web_material_uhae328.zip › Supplemetary Figure6.pdf]

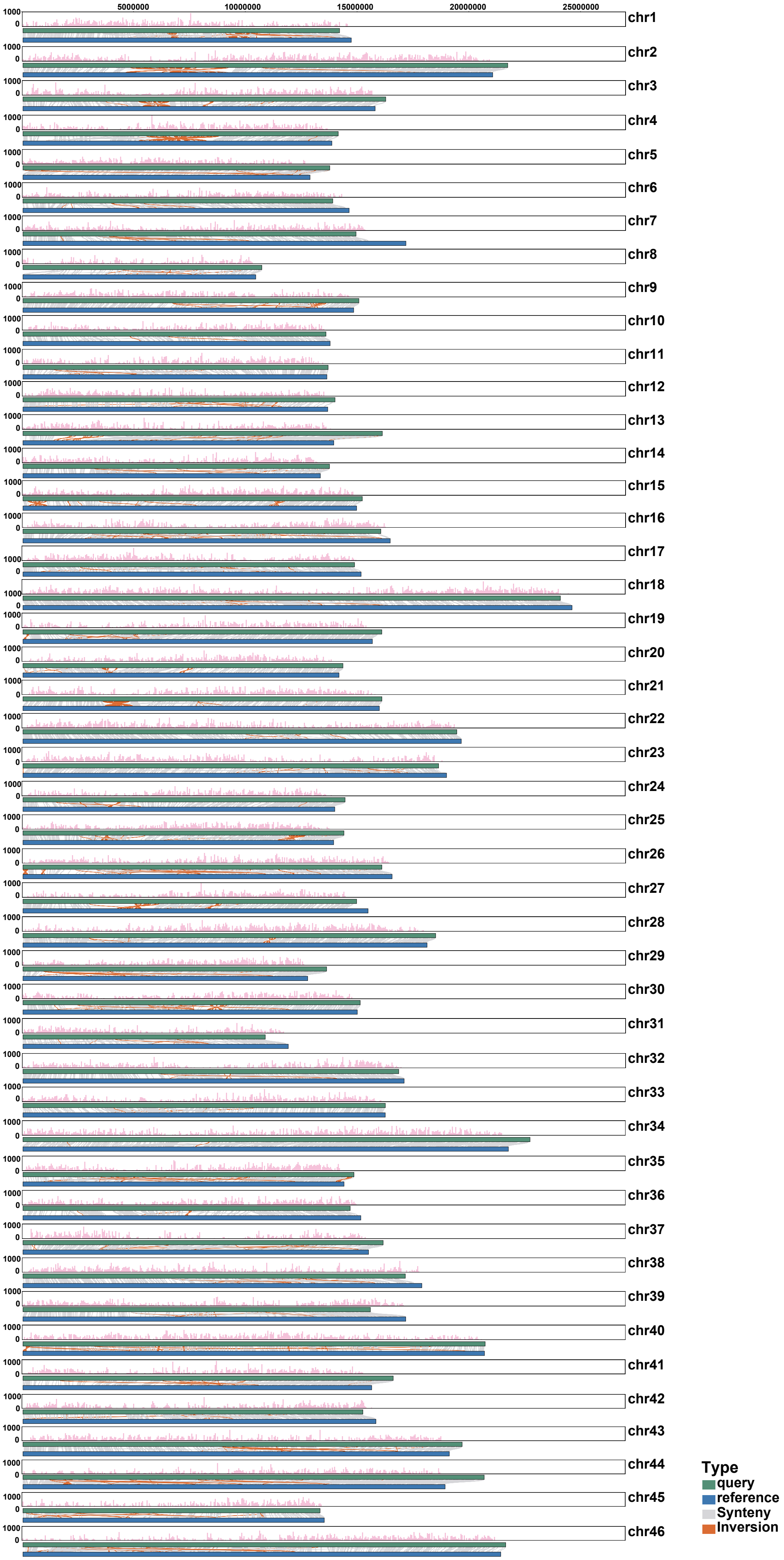

Fig. S6 Heterozygosity and synteny between homologous chromosomes.
The upper histogram shows the number of heterozygous SNP detected between homologous haplotypes. Lower bars represent the two haplotypes of *S. ningpoensis* chromosomes; gray lines indicate the aligned sequences between the haplotypes.
