# Supplementary material for: A chromosome-scale genome assembly and epigenomic profiling reveal temperature-dependent histone methylation in iridoid biosynthesis regulation in Scrophularia ningpoensis
Source: Hortic Res. 2025 Mar 4;12(3):uhae328. doi: 10.1093/hr/uhae328 (PMC11879554; doi:10.1093/hr/uhae328)
Supplement: Web_Material_uhae328 [file web_material_uhae328.zip › Supplemetary Figure8.pdf]

A

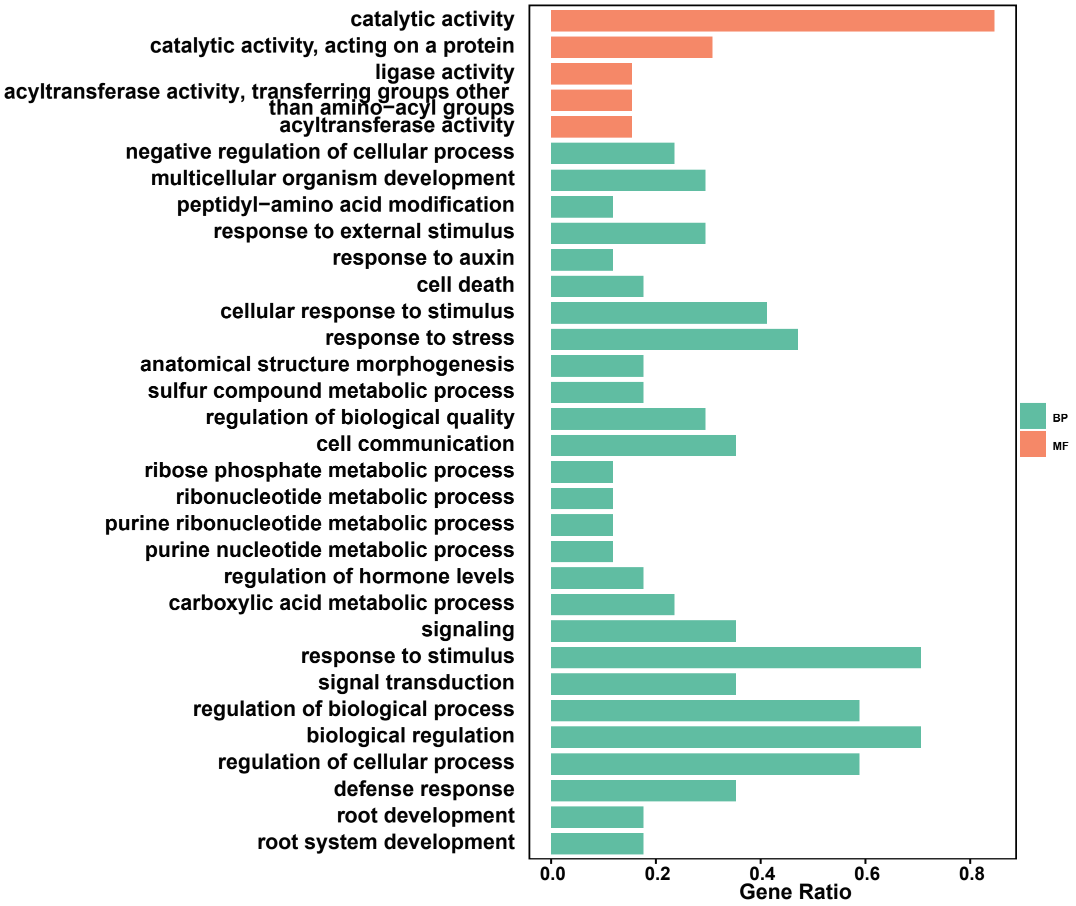

B

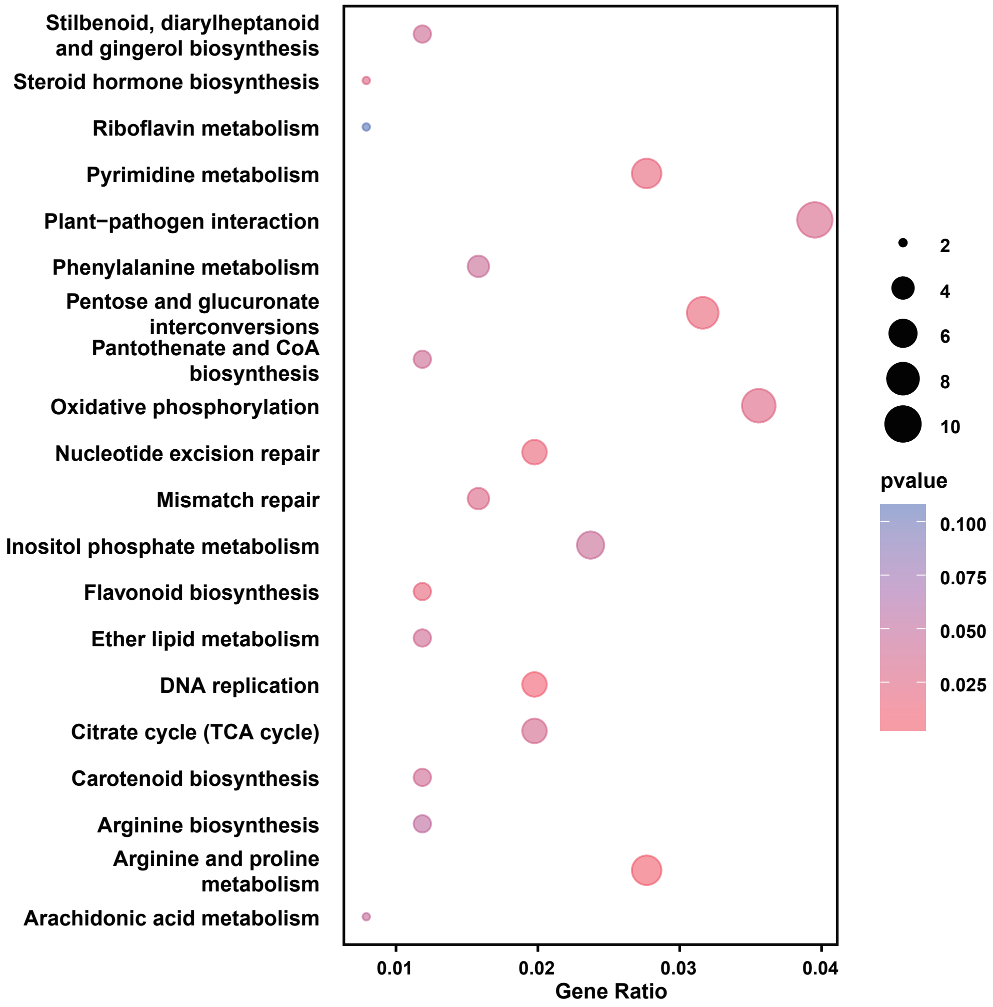

Fig. S8 GO/KEGG enrichment analysis of the genes with ASEGs.
(A) GO enrichment analysis of the genes with ASEGs. **(B)** KEGG enrichment analysis of the genes with ASEG.
