# Supplementary figures and images for: A chromosome-scale genome assembly and epigenomic profiling reveal temperature-dependent histone methylation in iridoid biosynthesis regulation in Scrophularia ningpoensis
Source: Hortic Res. 2025 Mar 4;12(3):uhae328. doi: 10.1093/hr/uhae328 (PMC11879554; doi:10.1093/hr/uhae328)

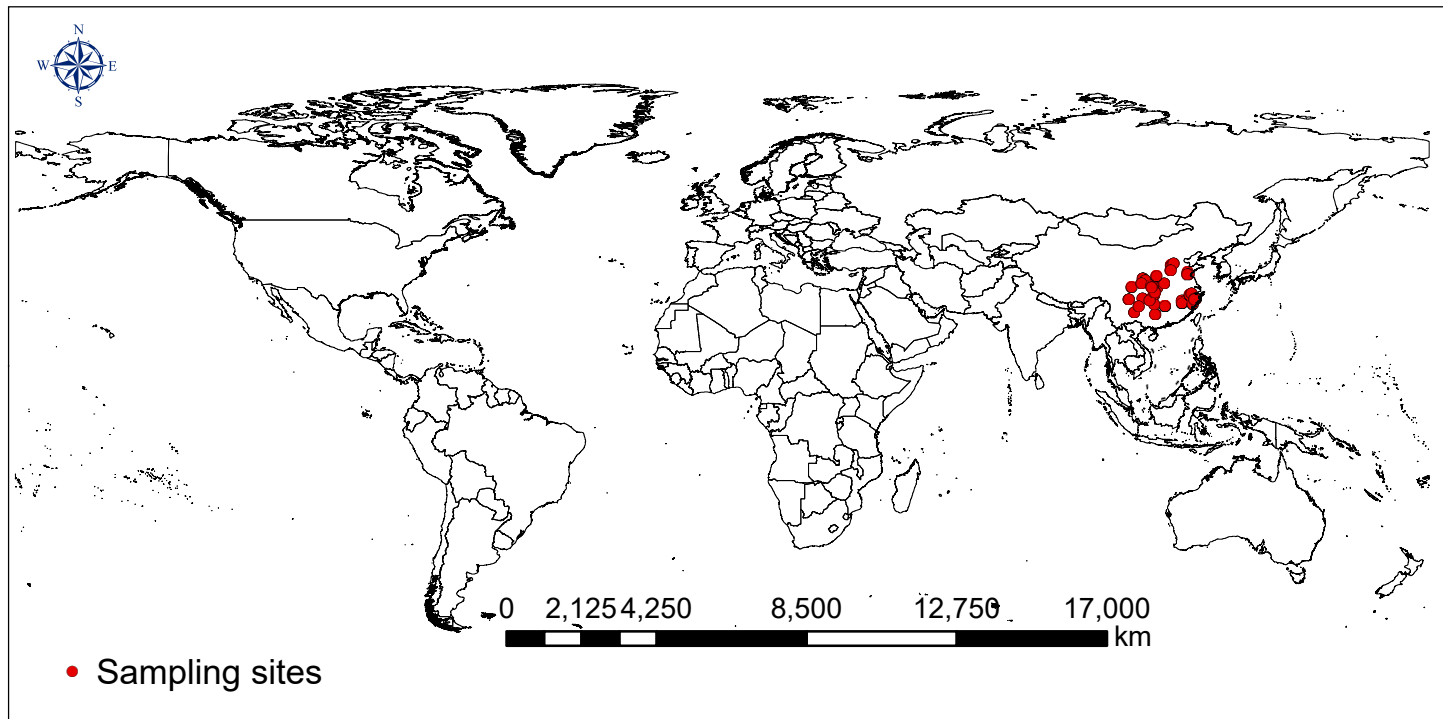

Fig. S1 Global sampling sites of *S. ningpoensis*.

Supplement: Web_Material_uhae328 [file web_material_uhae328.zip › Supplemetary Figure1.pdf]

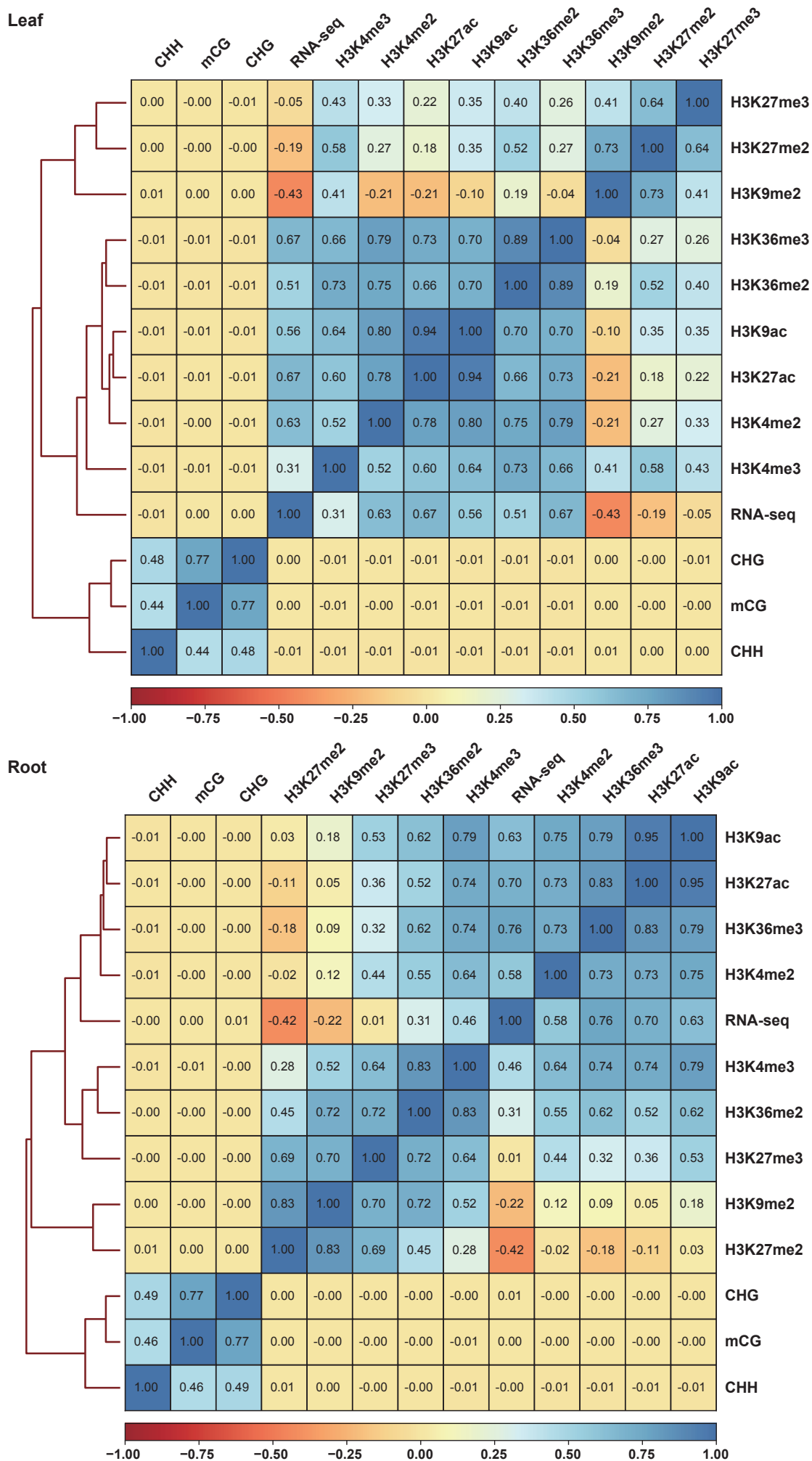

Fig. S15 Dynamics of histone marks and gene expression in the two tissues of *S. ningpoensis*.

Supplement: Web_Material_uhae328 [file web_material_uhae328.zip › Supplemetary Figure15.pdf]

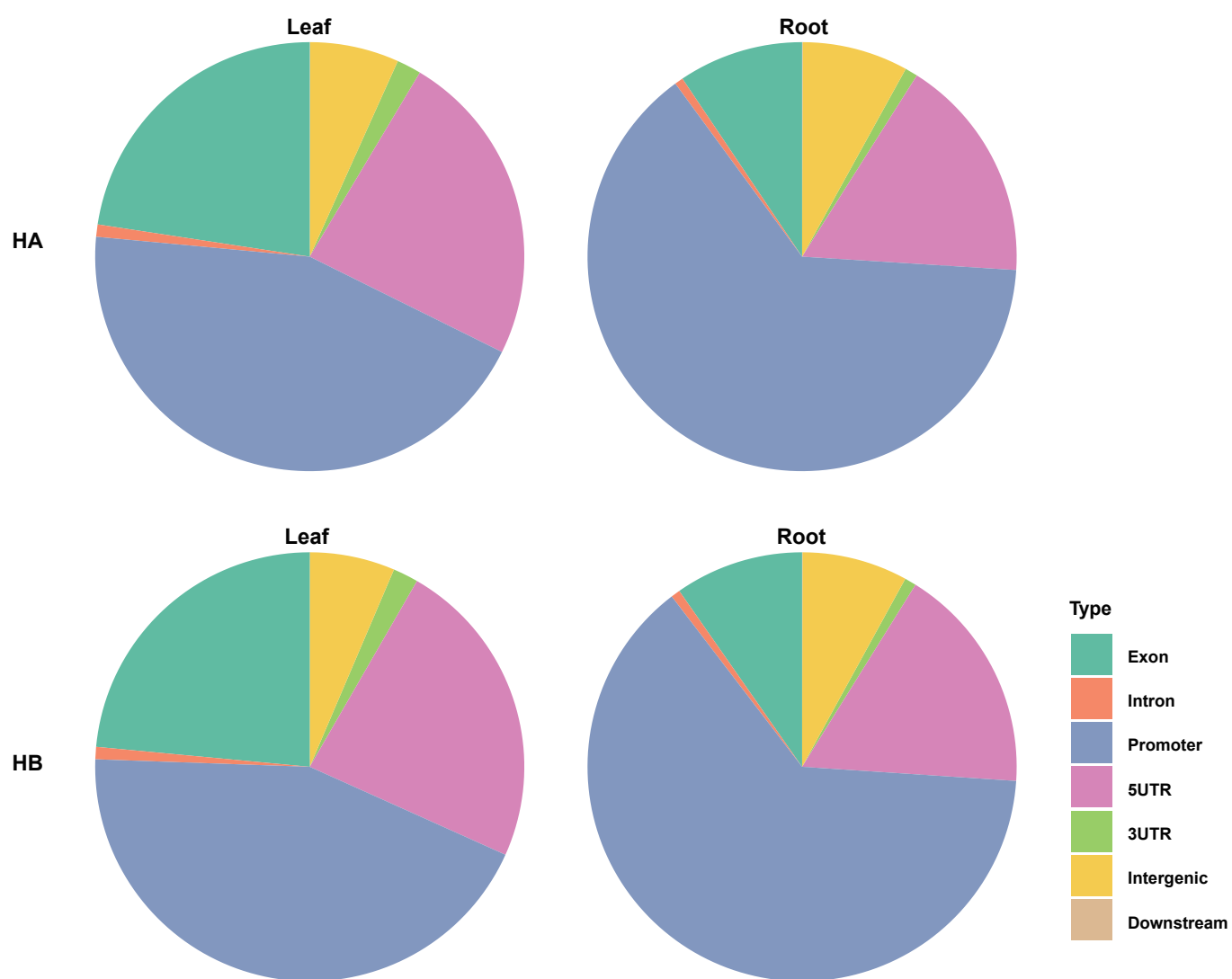

Fig. S17 Genic distributions of H3K36me3 and their relationship to ASEG gene expression.

Supplement: Web_Material_uhae328 [file web_material_uhae328.zip › Supplemetary Figure17.pdf]

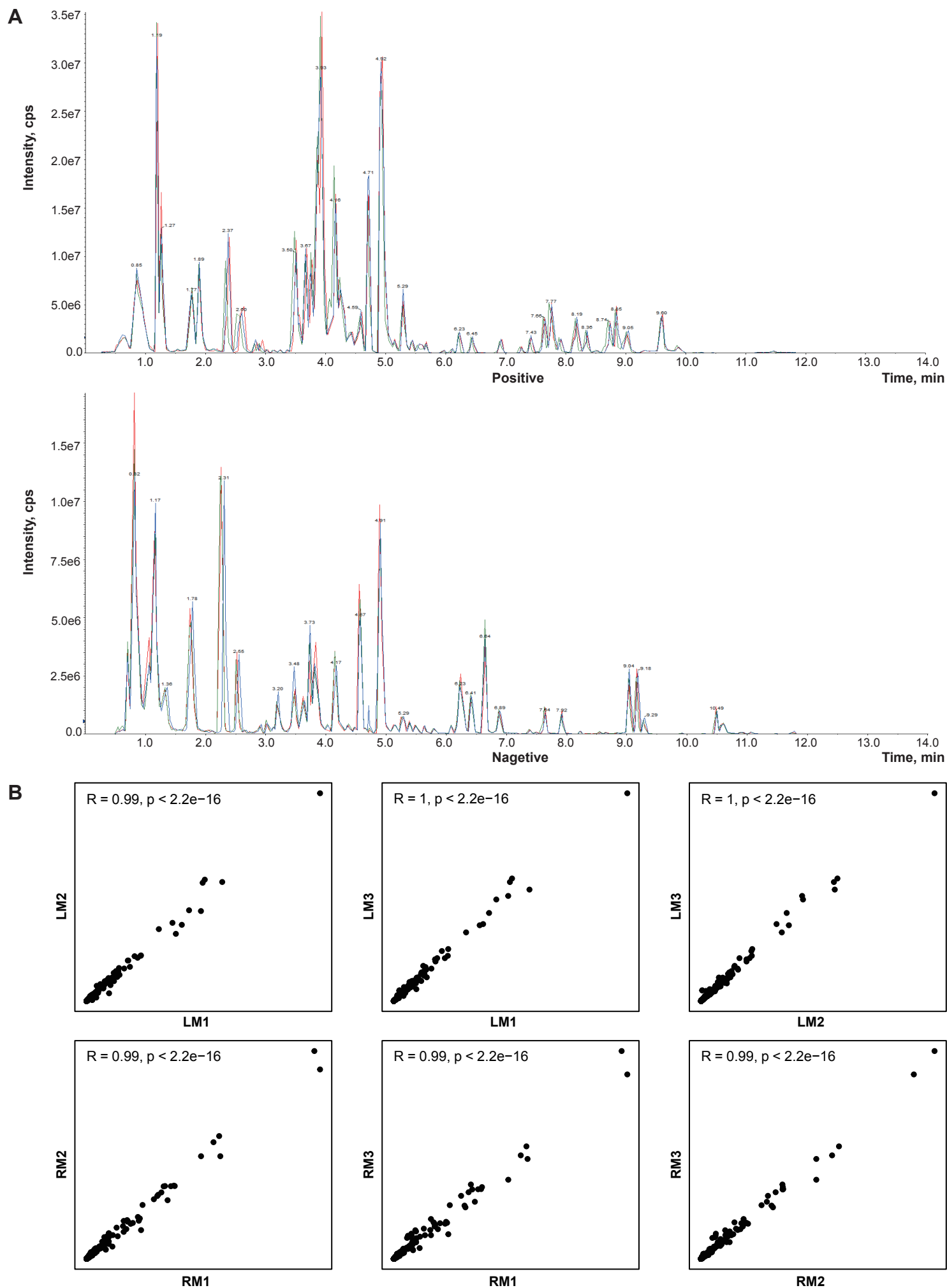

Supplement: Web_Material_uhae328 [file web_material_uhae328.zip › Supplemetary Figure18.pdf]

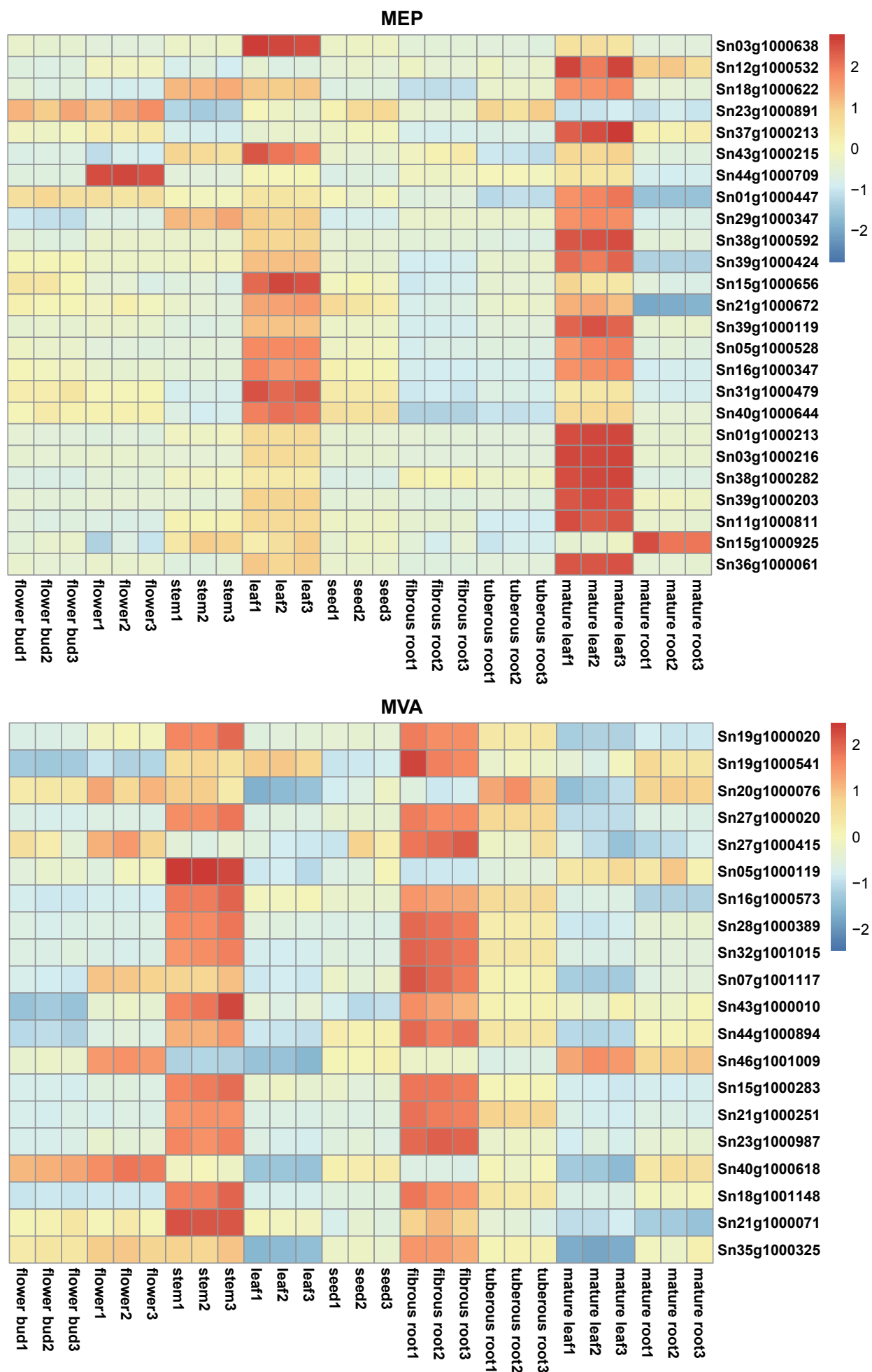

Fig. S21 Expression pattern of iridoids related genes across nine tissues in *S. ningpoensis*.

Supplement: Web_Material_uhae328 [file web_material_uhae328.zip › Supplemetary Figure21.pdf]

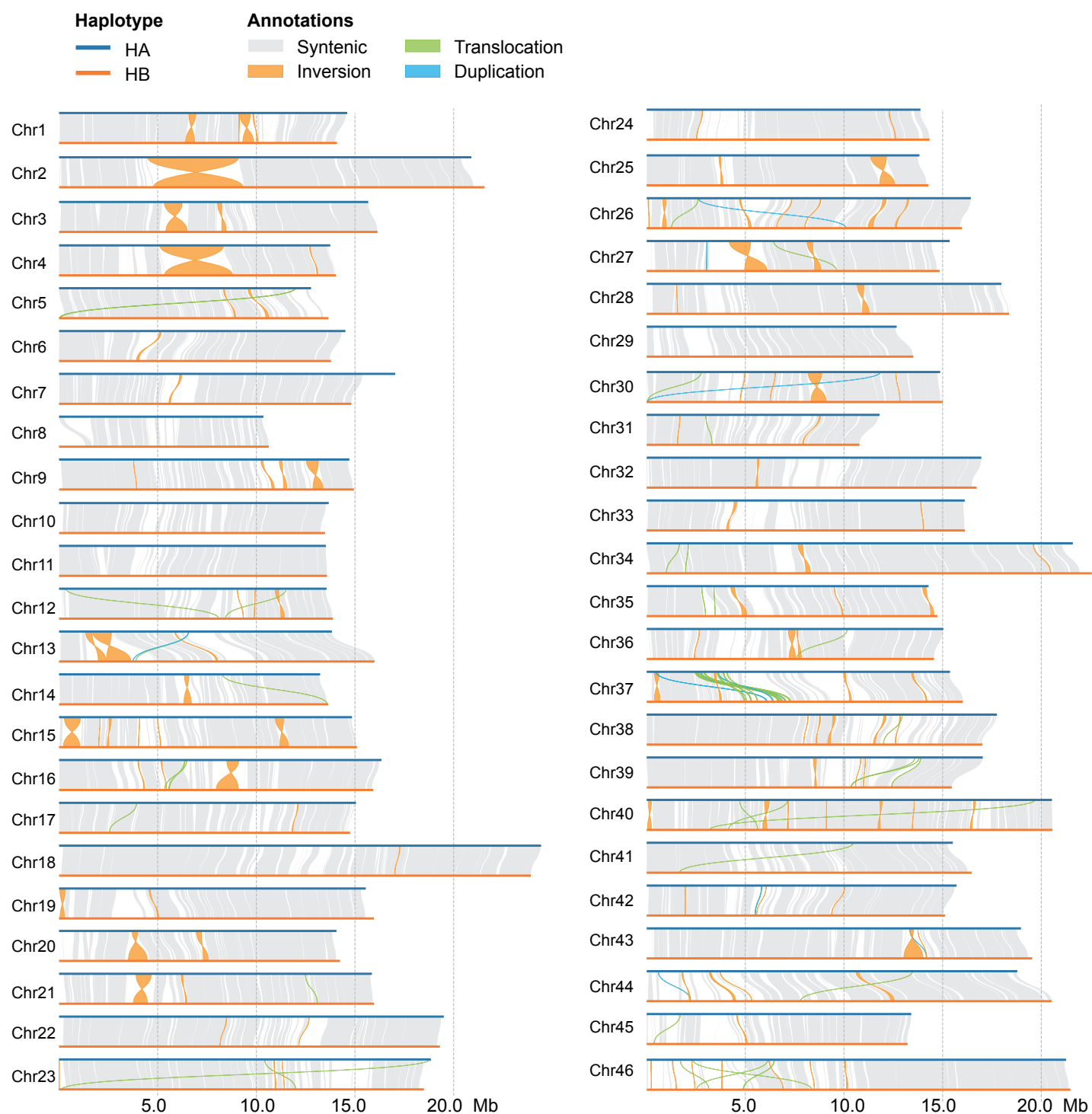

**Fig. S7 Visualization of structural variation between allelic chromosome pairs.**

Supplement: Web_Material_uhae328 [file web_material_uhae328.zip › Supplemetary Figure7.pdf]

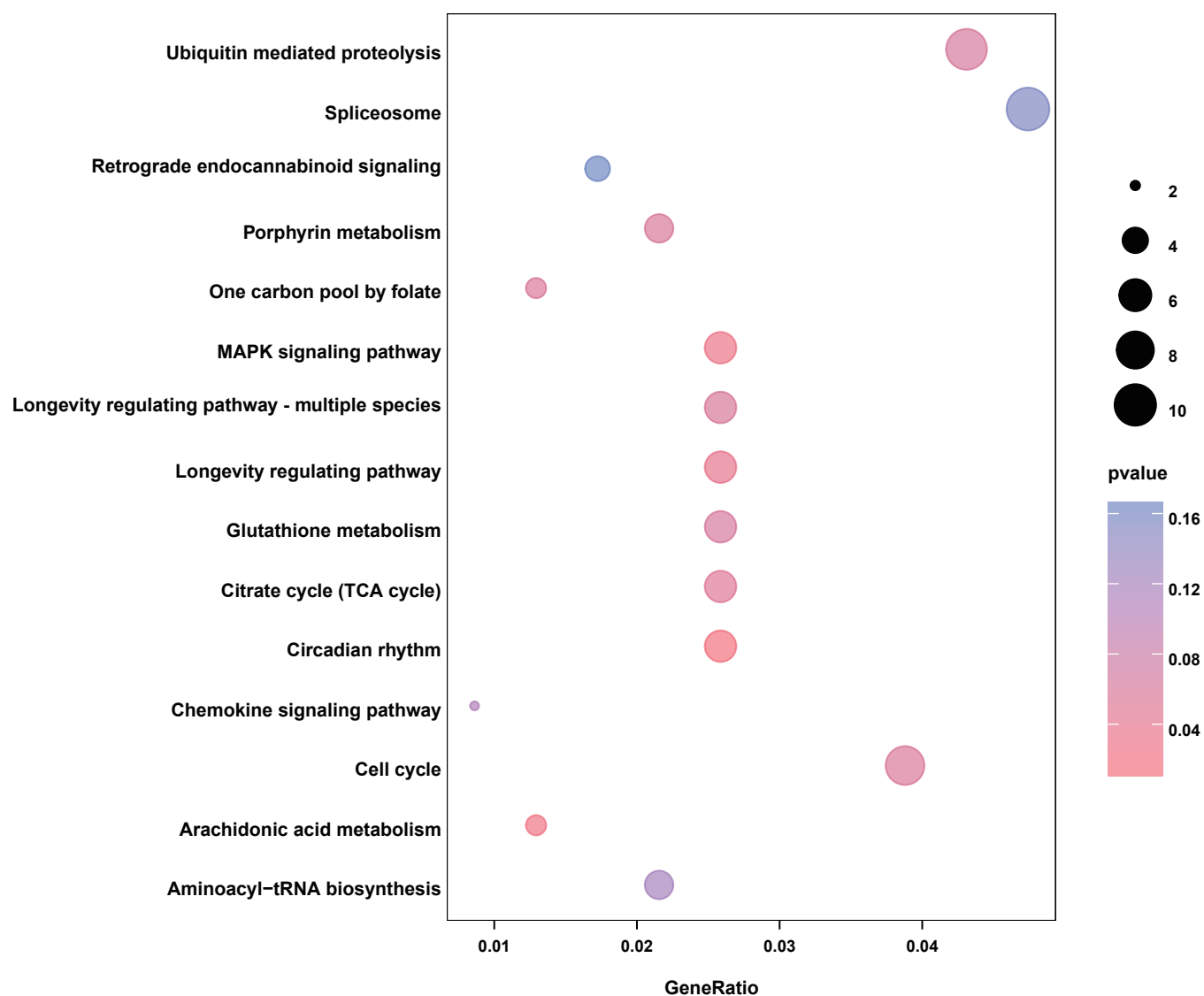

Fig. S9 KEGG enrichment analysis of genes with large-effect variations.

Supplement: Web_Material_uhae328 [file web_material_uhae328.zip › Supplemetary Figure9.pdf]
